# Cellular response of advanced triple cultures of human osteocytes, osteoblasts and osteoclasts to high sulfated hyaluronan (sHA3)

**DOI:** 10.1016/j.mtbio.2024.101006

**Published:** 2024-02-22

**Authors:** Katharina Wirsig, Jana Bacova, Richard F. Richter, Vera Hintze, Anne Bernhardt

**Affiliations:** a Centre for Translational Bone, Joint- and Soft Tissue Research, Faculty of Medicine and University Hospital, TUD University of Technology, Fetscherstraße 74, 01307, Dresden, Germany; b Max Bergmann Center of Biomaterials, Institute of Material Science, TUD University of Technology, Budapester Str. 27, 01069, Dresden, Germany; c Department of Biological and Biochemical Sciences, Faculty of Chemical Technology, University of Pardubice, Studentska 573, 53210 Pardubice, Czech Republic

**Keywords:** Triple culture, Osteoblast, Osteocyte, Osteoclast, BMP-2, sHA3

## Abstract

Bone remodelling, important for homeostasis and regeneration involves the controlled action of osteoblasts, osteocytes and osteoclasts. The present study established a three-dimensional human *in vitro* bone model as triple culture with simultaneously differentiating osteocytes and osteoclasts, in the presence of osteoblasts. Since high sulfated hyaluronan (sHA3) was reported as a biomaterial to enhance osteogenesis as well as to dampen osteoclastogenesis, the triple culture was exposed to sHA3 to investigate cellular responses compared to the respective bone cell monocultures. Osteoclast formation and marker expression was stimulated by sHA3 only in triple culture. Osteoprotegerin (OPG) gene expression and protein secretion, but not receptor activator of NF-κB ligand (RANKL) or sclerostin (SOST), were strongly enhanced, suggesting an important role of sHA3 itself in osteoclastogenesis with other targets than indirect modulation of the RANKL/OPG ratio. Furthermore, sHA3 upregulated osteocalcin (BGLAP) in osteocytes and osteoblasts in triple culture, while alkaline phosphatase (ALP) was downregulated.

## Highlights

• Expression of OCy-markers (MEPE, SOST, DMP1) is upregulated by BMP-2.
• OCy and OC can be differentiated simultaneously in triple culture with OBs.
• sHA3 stimulates OC-formation in triple culture but not in monoculture.
• TRAP activity of OC is enhanced by sHA3, whereas resorption is inhibited.
• BGLAP is upregulated in response to sHA3, whereas ALP is downregulated.

## Graphical abstract

**Figure:**
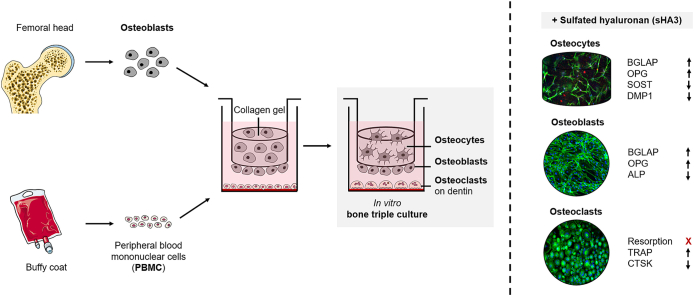

## 1 Introduction

Bone tissue undergoes a constant remodelling process mediated by bone formation of osteoblasts (OBs) and bone resorption of osteoclasts (OCs). Matrix-embedded osteocytes (OCys) act as mechanosensors and control bone turnover mechanisms [1]. Important signalling factors for OC-differentiation are receptor activator of NF-κB ligand (RANKL) and macrophage colony-stimulating factor (MCSF), mainly secreted by OBs and OCys, inducing the fusion of mononuclear progenitor cells [2]. The use of animal models to study mechanisms of physiological cellular processes in bone is limited. They do not have the biological programmes specific to the human species and are therefore poorly predictive for clinical outcomes. Therefore, we aimed to establish a bone model consisting exclusively of primary human cells, reducing the need for complex and costly animal models. A recently developed triple culture as *in vitro* bone model with primary human bone cells [3] allowed studies on the crosstalk between OBs, OCys and OCs, first differentiated in monocultures and then brought together in a triple culture. OBs were isolated from human femoral heads and differentiated into OCys by embedding them into collagen gel in transwell inserts. OCs were obtained by differentiation of monocytes isolated from human buffy coat [4]. Effects on cellular behaviour of cells in triple culture were observed in response to extracts of magnesium degradation products [5], strontium ions or hypoxic cultivation conditions [6]. However, differentiation processes could not be investigated in this triple culture due to different media requirements for OCys (low serum) and OCs (10 % serum) differentiation. To mimic the bone remodelling process as closely as possible, OCys and OCs should be differentiated from their progenitor cells in the presence of the other bone cell types, providing insights into bone metabolism, a better understanding of cell communication during bone remodelling, as well as the evaluation of drugs and biomaterial extracts in an environment close to human native bone tissue. To date, bone replacement materials capable of releasing biologically active ions and molecules have often been studied only with OBs, their progenitor cells or cell lines. To our knowledge, there is still a lack of studies focusing on cellular behaviour in co-cultures of bone cells comprising all the three cell types or their precursors. To understand the effects of bioactive molecules or different cultivation conditions, it is worth investigating not only the interaction between bone-forming OBs and bone-resorbing OCs, but also the influence on OCys that orchestrate bone remodelling via key signalling pathways [7,8] such as Wnt/β-catenin [9] or notch pathway [10].

Bone tissue consists of both inorganic and organic components. The inorganic part, consisting mainly of crystalline hydroxyapatite, provides hardness and resistance to mechanical stress. Flexibility and elasticity are provided by the organic phase, comprising a range of proteins with type I collagen (∼90 %) being the most abundant and major structural component [11,12]. Non-collagenous proteins, including extracellular matrix (ECM) proteins, growth factors, cytokines and lipids compose the remaining organic material and facilitate biological functions such as bone matrix mineralisation or regulation of OB- and OC-function and differentiation [1,13]. Glycosaminoglycans (GAGs) are also part of the organic ECM [14]. Their negative charges allow the binding of growth factors, cytokines and chemokines, thereby enabling the interference with signal transduction cascades. Sulfation of GAGs modulating extracellular signals such as cell-cell and cell-ECM interactions can occur at various positions within the glycan backbone. GAGs are involved in numerous signalling cascades in bone formation and development. The degree of sulfation of GAGs is highly specific in relation to their function [15]. Changes in sulfation patterns can lead to alterations and imbalances in signalling pathways, thereby affecting skeletal development [16]. GAGs like hyaluronan (HA) and its artificial modifications via sulfation (sHA) are potential candidates for the induction of cellular responses in bone tissue and for the functionalisation of bone graft materials. Studies have shown that high sulfated HA (sHA3) improves bone defect regeneration of diabetic rats by enhancing OB-function and sHA3's high binding affinity to sclerostin (a molecule involved in OC differentiation as potent inhibitor of Wnt signalling and OB-function) [17]. Furthermore, this study demonstrated that sHA3 induced bone morphogenetic protein 2 (BMP-2), lowered the RANKL/osteoprotegerin (OPG) expression ratio and decreased the differentiation of OCs. Partially, contrary effects of sHA were observed on the differentiation and activity of OCs: While synthetically derived highly sulfated HA suppressed the formation of OCs and inhibited resorption [18], another study recently reported an upregulation of TRAP activity in response to sHA3 for OC-monocultures derived from peripheral blood mononuclear cells (PBMCs) isolated from diabetic patients with charcot neuropathy [19]. In addition Schulze et al. found a significantly reduced tissue nonspecific alkaline phosphatase (TNAP) activity of OBs treated with sHA3. Further studies on the impact of sHA3 on bone cells, especially in co-cultures are needed to answer open questions of cellular mechanisms.

In the present study, we aim for the first time to reduce the amount of supplements in a triple culture medium for OBs and progenitors of OCys and OCs to a minimum, enabling differentiation processes in an *in vitro* bone model by cellular signalling and not by externally added substances. Therefore, a) a medium composition was evaluated supporting the differentiation of both OCy and OC from their respective progenitors. In addition, b) freshly isolated PBMC as well as cryopreserved ones were tested for their potential to differentiate into mature OCs in monoculture and triple culture. Finally c) the impact of sHA3 on cellular behaviour in the established human bone triple culture over a time period of 14 days was tested in comparison to monocultures of OBs, OCys and OCs. Cell morphology, marker gene expression and enzyme activities by the respective cell types as well as resorption of OCs on dentin and secretion of BGLAP, SOST, RANKL and OPG in cell culture supernatants were investigated.

## 2 Materials and methods

### 2.1 Cell culture

#### 2.1.1 Osteoblasts and differentiation of osteocytes

**Fig. 1:**
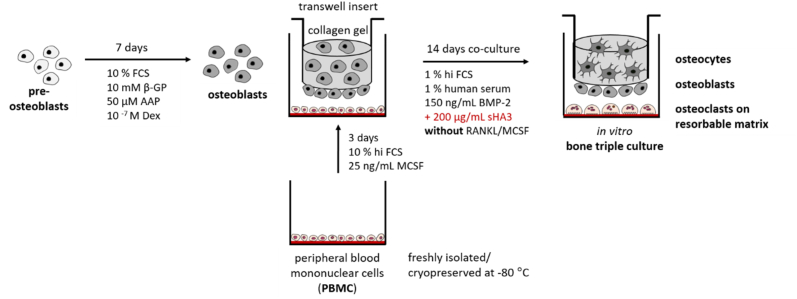
Experimental setup and cultivation conditions for the preparation of *in vitro* triple cultures of OBs and simultaneously differentiating OCys and OCs from freshly isolated and cryopreserved PBMC. β-glycerophosphate (β-GP), ascorbic acid-2-phosphate (AAP), dexamethasone (Dex), heat inactivated FCS (hi FCS), bone morphogenetic protein 2 (BMP-2), high sulfated hyaluronan (sHA3), receptor activator of NF-κB ligand (RANKL), macrophage colony stimulating factor (MCSF).

Primary human pre-OBs were isolated from human femoral heads of osteoarthritic patients undergoing total hip replacement at the University Hospital *Carl Gustav Carus* Dresden (Germany) after informed consent (approval by the ethics commission of TU Dresden, EK 303082014, date of approval: 08.08.2014) as previously described [20]. OBs were expanded in α-MEM with GlutaMAX (Gibco) containing 15 % FCS, 100 U/mL penicillin and 100 μg/mL streptomycin (PS) (Gibco). For differentiation into OBs, pre-OBs were cultured for 7 days in osteogenic medium (α-MEM with GlutaMAX containing 10 % FCS, PS, 10^−7^ M dexamethasone (Dex), 10 mM β-glycerophosphate (β-GP) and 12.5 μg/mL ascorbic acid-2-phosphate (AAP) (Fig. 1), all osteogenic supplements were purchased from Sigma-Aldrich).

For OCy-differentiation human pre-OBs were cultivated in osteogenic medium for 7 days, then harvested with Trypsin/EDTA (Gibco) and embedded in collagen gels as previously published [3]. Briefly, 8 parts of collagen solution (4 mg/mL rat tail collagen, Meidrix Biomedicals GmbH) were mixed with one part of 10x HBSS, 10 mM β-GP, 12.5 mM AAP and neutralized with 1 N NaOH. Cells were resuspended in this mixture in a final concentration of 1•10^5^ cells/mL. Each 500 μL of this collagen/cell suspension was pipetted into 24-well plates for OCy-monocultures. Collagen gelation was performed in an incubator at 37 °C for 30 min. After the collagen had fibrillated, different low serum media (FCS, heat inactivated FCS (hi FCS), human serum (HS), insulin transferrin selenium (ITS)) based on α-MEM with GlutaMAX containing PS, were added (Table 1). Cells in collagen gels were incubated for 14 days to complete the differentiation of OCys from OBs, with one medium change after 7 days, as previously described [21].

**Table 1:** Media compositions for OCy-differentiation.

osteocyte medium	serum and supplements
1	2 % FCS, 10 mM β-GP, 12.5 μg/mL AAP
2	1 % ITS, 10 mM β-GP, 12.5 μg/mL AAP, 33,3 μg/mL BMP-2
3	1 % ITS, 10 mM β-GP, 12.5 μg/mL AAP, 33,3 μg/mL BMP-2 + 10 nM VitD3
4	1 % ITS, 10 mM β-GP, 12.5 μg/mL AAP, 100 μg/mL BMP-2
5	1 % hi FCS, 1 % HS
6	1 % hi FCS, 1 % HS, 50 ng/mL BMP-2
7	1 % hi FCS, 1 % HS, 100 ng/mL BMP-2
8	1 % hi FCS, 1 % HS, 150 ng/mL BMP-2

#### 2.1.2 Differentiation of osteoclasts

OCs were differentiated from PBMC which were isolated from human buffy coat of six different donors (purchased from German red Cross, Dresden) by density gradient centrifugation as previously described [22]. Both freshly isolated and cryopreserved PBMC were tested for their ability to differentiate into multinucleated OCs under low serum conditions. PBMC in monoculture differentiate into multinucleated OCs in the presence of 25 ng/mL macrophage colony stimulating factor (MCSF) and 50 ng/mL RANKL (both from Peprotech). In some cases (see 3.2.), co-cultures of PBMC with OBs were used to analyse the impact of different media on OC-formation without the need to supply the cultures with additional RANKL. In that case, PBMC (1•10^6^) were cultivated in transwell inserts and hOB (4•10^4^) were seeded onto the corresponding 24-well plate. Media were based on α-MEM with GlutaMAX containing PS, MCSF, RANKL and different amounts of serum or serum alternatives (Table 2). Platelet lysate (PL) was prepared from expired platelet concentrates by 4 freeze/thawing cycles followed by sterile filtration. Concentrates of 5 different donors were pooled. For OC-monoculture, PBMC containing 5•10^5^ monocytes, were seeded into 48 well plates. Cells were cultured one day in α-MEM with GlutaMAX containing PS, 10 % hi FCS and 25 ng/mL MCSF. Afterwards medium was changed to OC-medium 1–7 (Table 2), containing additionally 50 ng/mL RANKL for 6 days with one medium change in between.

**Table 2:** Media compositions for OC-differentiation.

osteoclast medium	serum and supplements
1	5 % hi FCS, 5 % HS
2	1 % hi FCS, 1 % HS
3	1 % hi FCS, 1 % HS, 150 ng/mL BMP-2
4	2 % HS
5	2 % PL
6	1 % ITS
7	1 % ITS, 150 ng/mL BMP-2

To freeze and thaw PBMC, cells were cryopreserved in the presence of MCSF which is secreted by the bladder carcinoma cell line 5637. In addition to MCSF, cell line 5637 secretes other cytokines such as granulocyte colony stimulating factor (GCSF), granulocyte macrophage colony stimulating factor (GMCSF), stem cell factor (SCF) and interleukin-1β (IL-1β) [23]. For enrichment of MCSF in the cell culture supernatant, 5637 cells were grown to confluence in α-MEM with glutamax containing PS and 20 % hi FCS followed by a medium change with 10 mL per T75 cell culture flask and five days of cultivation without media change. The MCSF-containing supernatant was removed, sterile filtered and stored at −80 °C. PBMC were frozen at a density of 2•10^7^ PBMC/200 μL freezing medium (50 % hi FCS, 40 % MCSF-containing supernatant, 10 % DMSO) at −80 °C.

#### 2.1.3 Resorbable matrix

To analyse the resorption capacity of OCs *in vitro*, not only passively by measuring osteoclastic enzyme activities, but also directly by the formation of resorption pits, resorbable matrices in cell culture are required. Therefore PBMC were seeded on dentin discs and differentiated into mature OCs. Dentin slices were prepared from canine teeth of minipigs. Teeth were sawed into discs of 0.8 mm thickness using an electric diamond saw. After disinfection with ethanol for at least 1 h, dentin discs were placed in 24-well plates, air dried and washed once with PBS, before seeding with PBMC.

#### 2.1.4 Triple cultures with osteoblasts and simultaneously differentiating osteocytes and osteoclasts from their respective precursors

*In vitro* bone triple cultures of collagen-gel embedded OBs (differentiating to OCys), OBs and PBMC (differentiating to OC) were performed in transwell inserts (0.4 μm pore size; Sarstedt) in 24-well plates (Fig. 1). First, OBs were seeded with a density of 2•10^4^/membrane on the apical side of transwell inserts. Cells were allowed to attach to the polyethylene terephthalate (PET) membrane for 1 h in the incubator without further medium supply. Afterwards, primary human OBs (four donors, see Table 4), mixed into 250 μL of neutralized collagen solution (1•10^5^ OBs/mL) were transferred to the basal side of the same transwell inserts. Collagen gelation was performed in an incubator at 37 °C for 30 min. Constructs were then placed in 24-well plates, pre-seeded (3 days) with freshly isolated or cryopreserved PBMC (1,3•10^6^ per well, see 2.1.2.). PBMC were cultured directly on tissue culture polystyrene (TCPS) or dentin discs and cultured in α-MEM with GlutaMAX containing 10 % hi FCS, PS and 25 ng/mL MCSF until triple culture assembly. After building up triple cultures, medium that supports both, OCy-differentiation and OC-formation in monocultures (α-MEM with GlutaMAX containing 1 % hi FCS, 1 % HS, PS, 150 ng/mL BMP-2, see 3.1 and 3.2.) was added to the constructs (1 mL in the well plate, 500 μL to the transwell insert). Besides this triple culture medium, triple cultures as well as OB/OCy/OC monocultures were cultured in the presence of 200 μg/mL synthetically derived sHA3 (provided by Dr. S. Möller and Dr. M. Schnabelrauch, Innovent e.V. Jena) in an incubator with controlled O_2_ content of 21 % and 5 % CO_2_ for 14 days (Fig. 1). Medium was partially changed (500 μL) after 7 days in triple cultures and after every 3–4 days in monocultures.

#### 2.1.5 Sources of osteoblasts/osteocytes and PBMC

OCy-monocultures were analysed in five independent experiments, involving OBs/OCys of five different donors. Donor information for human pre-OBs is given in Table 3. Triple cultures of OBs, OCys and OCs with simultaneous differentiation of OCys and OCs were analysed in five independent experiments, including OBs/OCys of five different donors and OCs derived from PBMC of four different donors (Table 4). Information on age and sex of the blood donors was not provided by the German Red Cross.

**Table 3:** Donor information for primary human pre-OBs, which were used for the differentiation of OBs/OCys in five independent experiments.

experiment no.	osteoblasts/osteocytes
1	female 56 years
2	female 58 years
3	male 61 years
4	male 57 years
5	male 62 years

**Table 4:** Donor information for primary human pre-OBs, which were used for the differentiation of OBs/OCys and combined PBMC donors for five independent triple culture experiments investigating the impact of freshly isolated versus cryopreserved PBMC or the impact of sHA3.

experiment no.	osteoblasts/osteocytes	osteoclasts	fresh/cryopreserved	sHA3
1	male 57 years	PBMC donor 1	X	
2	male 62 years	PBMC donor 2	X	X
3	male 62 years	PBMC donor 3	X	X
4	female 58 years	PBMC donor 4		X
5	male 61 years	PBMC donor 4		X

### 2.2 Fluorescence microscopy

Transwell insert membranes, seeded with OBs and OCy-containing collagen gels were removed from the inserts. Remaining 24 -well plates with PBMC/differentiated OCs as well as other samples were washed once with PBS and cells were fixed by incubation with phosphate buffered formaldehyde (4 %) for at least 1 h. After permeabilisation (0.1 % Triton X-100 in PBS for 5 min) and blocking (1 % BSA in PBS/30 min) OBs and OCs were incubated with DAPI (ThermoFisher Scientific) to detect cell nuclei and Phalloidin-iFluor 488 (Abcam) to visualise cytoskeleton (1 h protected from light). To observe specific biomarkers and morphological changes, OCys were stained for dentin matrix protein 1 (DMP1) and sclerostin (SOST). Primary and secondary antibodies were diluted in 1 % BSA in PBS. Rabbit anti-human DMP1 (TaKaRa; dilution 1:600; working concentration: 3.3 μg/mL) respectively rabbit anti-human SOST (antibodies-online GmbH; dilution 1:100; working concentration: 100 μg/mL) was detected with AlexaFluor 546-conjugated goat anti-rabbit secondary antibody (Abcam; 1:250). Secondary antibodies were added together with DAPI and Phalloidin-iFluor 488. Z-stack images were acquired using a Keyence BZ-X810 fluorescence microscope.

### 2.3 RNA isolation, cDNA synthesis and PCR

For RNA isolation, PET membranes were separated from transwell inserts using surgical knife and tweezers. Collagen gels containing OCys were removed from transwell inserts and incubated with collagenase II solution (3 mg/mL collagenase II in α-MEM, 10 % FCS, 2 mM L-glutamine, PS, 3 mM CaCl_2_) for 1 h at 37 °C. The digests were transferred to 15 mL tubes, washed with PBS, centrifuged and supernatant was discarded. For each experimental group, six triple culture samples were used to generate two RNA samples from membrane pieces with OBs, OCy-pellets and PBMC/OCs in 24-well plates using the commercially available peqGOLD MicroSpin Total RNA Kit (Peqlab).

For cDNA synthesis from RNA samples, the High-Capacity cDNA Reverse Transcription Kit (Applied Biosystems) was applied according to manufacturer's instructions.

Gene expression analysis was performed using qPCR reactions with the TaqMan Fast Advanced Master Mix (Applied Biosystems) and TaqMan Gene Expression Assays for the following genes: actin-β (ACTB), bone gamma-carboxyglutamate protein (osteocalcin, BGLAP), bone sialoprotein II (BSP II, IBSP), receptor activator of NF-κB Ligand (RANKL, TNFSF11), osteoprotegerin (OPG, TNFRSF11B), alkaline phosphatase (ALPL), podoplanin (PDPN), matrix extracellular phosphoglycoprotein (MEPE), dentin matrix protein 1 (DMP1), sclerostin (SOST), tartrate-resistant acid phosphatase (ACP5, TRAP), cathepsin K (CTSK) and carbonic anhydrase II (CA2) (Applied Biosystems), according to manufacturer's instructions. PCR was run with an Applied Biosystems 7500 fast Real-Time PCR system. Gene expression was normalized to the expression of ACTB (actin β) and relative gene expression (fold change) was calculated using the 2^−ΔΔCt^ method.

### 2.4 Quantification of enzyme activities

The activity of ALP for OBs and TRAP, CAII and CTSK activity for OCs were measured and normalized to the DNA content of the respective samples. Frozen membranes with OBs, respectively well plates with PBMC/OCs were thawed and cells were lysed by incubation in 1 % Triton X-100 in PBS for 50 min with an ultrasonication step for 10 min in between. Quantification of osteoclastic TRAP, CAII and CTSK activities was performed as previously described [24]. Briefly, ALP and CAII activities were determined calorimetrically by the cleavage of colourless p-nitrophenyl phosphate (ALP) and p-nitrophenyl acetate (CAII) respectively into yellowish p-nitrophenol in different buffer solutions and quantified by absorbance measurements at 405 nm using a spectrofluorometer infinite M200pro (Tecan Trading AG, Switzerland). TRAP activity was measured by cleavage of naphthol ASBI phosphate at acidic pH in the presence of tartrate and detection of the emitted fluorescence signals at an excitation and emission wavelength of 405/520 nm. CTSK activity was quantified by the cleavage of Z-LR-AMC (Enzo Life sciences) with fluorescence measurements at an excitation and emission wavelength of 365/440 nm. DNA concentration of cell lysates was quantified using Quantifluor One dsDNA kit (Promega) according to manufacturer's instructions.

### 2.5 TRAP staining

Samples were washed with PBS and fixed with 4 % formaldehyde in PBS for 15 min at room temperature. Fixation solution was removed by washing with PBS. TRAP staining was performed for 30 min at 37 °C with 0.3 mg/mL Fast Red Violet LB (Sigma-Aldrich) dissolved in an aqueous staining buffer containing 0.05 M sodium acetate (Sigma-Aldrich), 0.05 M acetic acid (Sigma-Aldrich), 0.03 M sodium tartrate (Roth), 0.1 mg/mL naphthol AS-MX phosphate disodium salt (Sigma-Aldrich) and 0.1 % Triton X-100 (Sigma-Aldrich). Afterwards, samples were washed with PBS and cell nuclei were stained for 10 min using Mayers Haemalaun solution (AppliChem) followed by rinsing in tap water. Bright field images of stained samples were taken using a Keyence BZ-X810 microscope.

### 2.6 ELISA-based detection of BGLAP, SOST, RANKL and OPG secretion

To determine the amount of BGLAP, SOST, RANKL and OPG protein secreted by OBs and OCys, the cell culture supernatants of the bone triple cultures and OCy-monocultures were analysed using solid phase sandwich enzyme-linked immunosorbent assay (ELISA) kits (Human Osteocalcin DuoSet ELISA # DY1419-05; Human TRANCE/RANKL/TNFS11 DuoSet ELISA # DY626; Human Osteoprotegerin/TNFRSF11B DuoSet ELISA # DY805; Human SOST/Sclerostin DuoSet ELISA # DY1406; all R&D Systems). After detection and development, absorbance measurements were performed at a wavelength of 450 nm with a wavelength correction at 540 nm. Protein concentrations were calculated by non-linear regression via recombinant human BGLAP (in the range of 312–10000 pg/mL)/SOST (62.5–4000 pg/mL)/RANKL (78.1–5000 pg/mL)/OPG (62.5–4000 pg/mL) calibration curves and normalisation to the total protein content quantified by bradford assay.

### 2.7 Resorption analysis

After performing cell lysis for biochemical measurements, dentin slices were washed with PBS and freeze-dried overnight. After drying, samples were mounted on sample holders, sputter coated with gold and imaged using a DSM 982 Gemini low-voltage scanning electron microscope with field emission gun (Zeiss, Germany) at 10/11 mm working distance, 6 kV and 200x magnification.

### 2.8 Statistics

Five experiments with different donors for OCy-differentiation were performed (Table 3, 3.1.). Formation of OCs from PBMC under low serum conditions was analysed in two experiments with different PBMC donors (3.2.). Five independent triple culture experiments were performed with cells of four OB/OCy donors in combination with four PBMC donors for the differentiation of OCs (Table 4). All samples of the individual experiments were seeded in triplicates for the measurement of enzyme activities and ELISA-based quantification of protein secretion in cell culture supernatant, while gene expression was analysed in two RNA samples generated from six triple culture replicates for each experiment. Statistical differences in gene expression were calculated on the level of ΔCt values. Normal (Gaussian) distribution of all data was analysed by Shapiro-Wilk normality test. One way ANOVA, followed by Dunnett's multiple comparison tests for normally distributed data and Kruskal-Wallis test, followed by Dunn's multiple comparison test for nonparametric data were used to compare more than two experimental groups (OCy-differentiation). Students t-test was carried out for the comparison of two experimental groups (triple culture fresh/cryopreserved PBMC, sHA3 treatment). GraphPad prism 9.0 was used for all statistical analyses.

## 3 Results

In an advanced triple culture model simultaneous OCy- and OC-differentiation are desirable. Therefore, different media compositions were tested with respect to their impact on both osteocyte and osteoclast differentiation to finally define a medium composition supporting the differentiation of both cell types.

### 3.1 Osteocyte differentiation

**Fig. 2:**
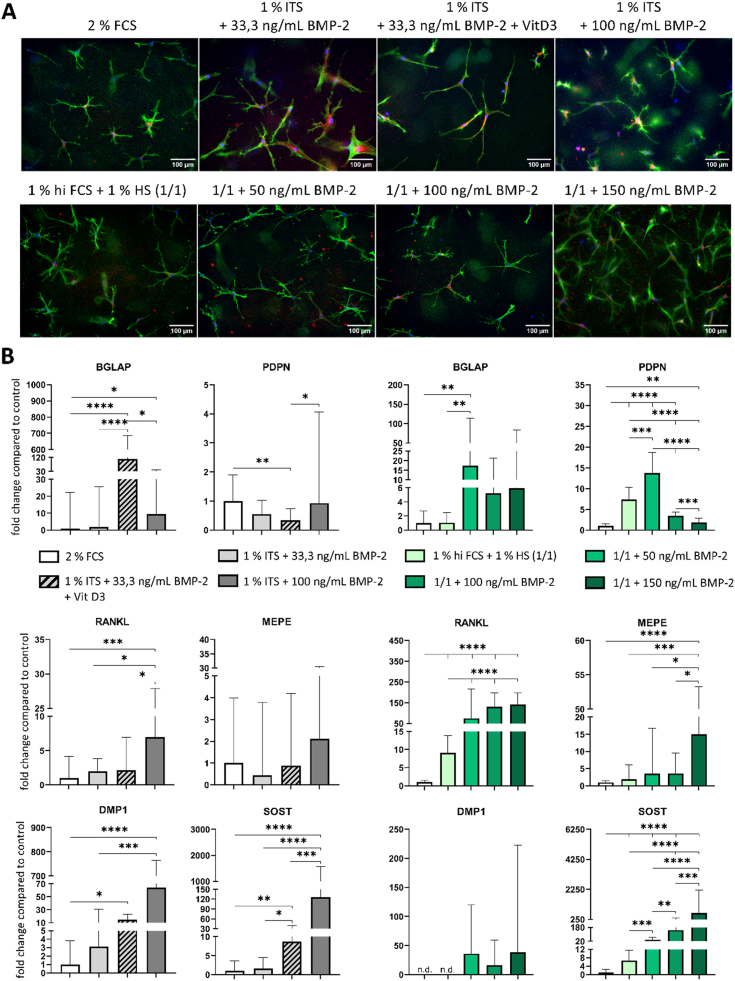
**Differentiation of collagen gel-embedded OCys cultivated over 14 days in different low serum containing media (FCS, ITS, hi FCS, HS) in the presence and absence of BMP-2 in concentrations between 33,3 ng/mL and 150 ng/mL. A**: Fluorescence microscopic images. Cytoskeleton appears green (iFluor488 phalloidin), nuclei appear blue (DAPI) and DMP1 appears red (Alexa546), scale bars represent 100 μm. **B**: Expression of OCy-marker genes BGLAP, PDPN, RANKL, MEPE, DMP1 and SOST. Diagrams show fold changes compared to 2 % FCS-based medium, ± upper and lower limit. High variances occur while combining three experiments for ITS-based media (grey, each experiment n = 6, n = 18 in total) and two experiments for hi FCS/HS based media (green, each experiment n = 6, n = 12 in total) due to variations between different donors. Therefore, gene expression data of single experiments are shown in Fig. S1. *p < 0.05; **p < 0.01, ***p < 0.001; ****p < 0.0001. (For interpretation of the references to colour in this figure legend, the reader is referred to the Web version of this article.)

Different media compositions were examined for OCy-differentiation in collagen gels (Table 1). In comparison to 2 % FCS containing OCy-medium, 1 % ITS containing medium with the addition of BMP-2 in different concentrations (33,3/100 ng/mL) and the combination of BMP-2 and vitamin D3 were tested. Moreover, OCy-medium containing 1 % hi FCS +1 % HS (1/1) with 50/100 or 150 ng/mL BMP-2 was investigated. OBs embedded in collagen gel differentiated into OCys with typical dendritic morphology in all tested media (Fig. 2A). Gene expression analysis revealed an upregulation of BGLAP in the presence of vitamin D3 and a strong significant upregulation of SOST by BMP-2. The highest expression of MEPE and SOST was detected in 1 % hi FCS +1 % HS containing medium with 150 ng/mL BMP-2 with significant difference to lower BMP-2 concentrations (Fig. 2B). RANKL expression was significantly upregulated by a BMP-2 concentration of 50 ng/mL or higher. The addition of BMP-2 ensured a stable expression of early and late OCy-markers.

### 3.2 Impact of low serum conditions on osteoclastic differentiation of PBMC

**Fig. 3:**
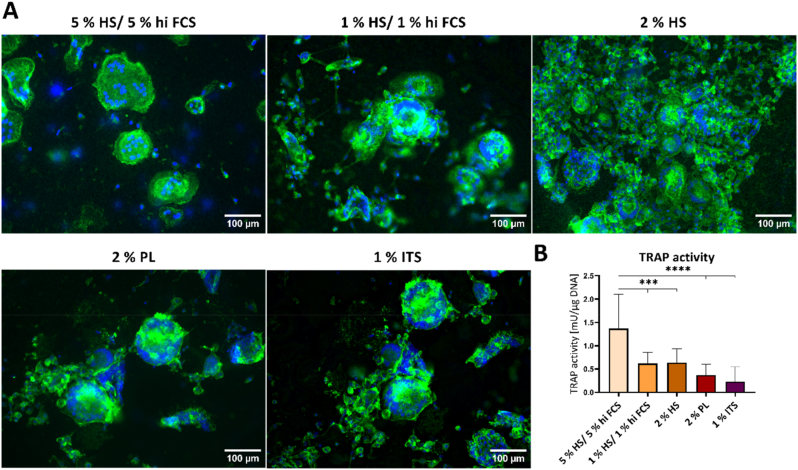
**Differentiation of OCs from freshly isolated PBMC in different low serum based media (hi FCS, HS, PL, ITS) in indirect co-culture with OBs. A**: Fluorescence microscopic images. Cytoskeleton appears green (iFluor488 phalloidin), nuclei appear blue (DAPI), scale bars represent 100 μm. **B**: TRAP activity. Diagram shows mean value ± standard deviations of two individual experiments (each n = 3, n = 6 in total). ***p < 0.001, ****p < 0.0001. (For interpretation of the references to colour in this figure legend, the reader is referred to the Web version of this article.)

Freshly isolated PBMC were differentiated into OCs in indirect co-culture with OBs under the addition of 1 % ITS, 2 % PL or decreased amounts of serum. Compared to the control (5 % HS/5 % hi FCS), OC-formation was reduced in all tested media (Fig. 3A). However, it was possible to generate multinucleated OC-like cells even in the presence of low serum or serum replacement. Activities of OC-specific TRAP demonstrated the functionality of generated OCs and were detected in all examined groups (Fig. 3B), lowest activities being identified in ITS supplemented media.

**Fig. 4:**
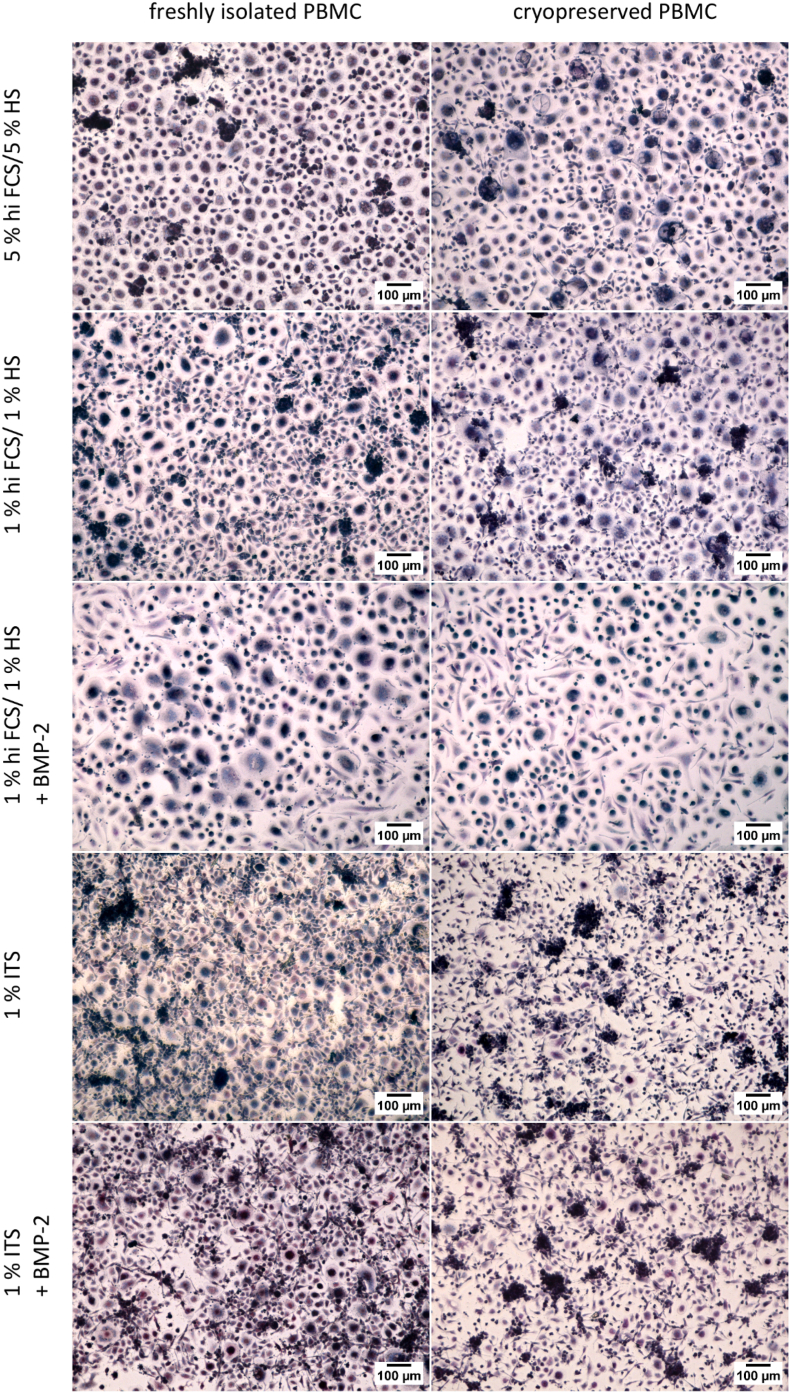
**TRAP staining of monocultured OCs derived from freshly isolated PBMC in comparison to cryopreserved PBMC in different low serum containing media (hi FCS/HS, ITS) with RANKL and MCSF and in the presence or absence of BMP-2.** Scale bars represent 100 μm.

Furthermore, freshly isolated PBMC as well as cryopreserved PBMC were differentiated into OCs in monoculture in the presence of RANKL and MCSF in different low serum or serum alternative based media. Osteoclastogenesis was also analysed in the presence of 150 ng/mL BMP-2, as OCy-differentiation requires the addition of BMP-2 for a stable OCy-marker expression (see 3.1., Fig. 2). TRAP-positive cells were observed in all examined groups, but multinucleated morphology of OC-like cells was more pronounced in serum-containing media compared to ITS-containing media (Fig. 4). Differentiation of OCs from their precursors and TRAP staining were not affected by supplementation with BMP-2. Furthermore, there were no differences in OC-morphology between freshly isolated and cryopreserved PBMC.

Conclusion from 3.1. and 3.2.: Optimal simultaneous OCy- and OC-differentiation can be realized by medium containing 1 % HS and 1 % hi FCS as well as 150 ng/mL BMP-2.

### 3.3 Triple culture with osteoblasts and simultaneously differentiating osteocytes and osteoclasts

**Fig. 5:**
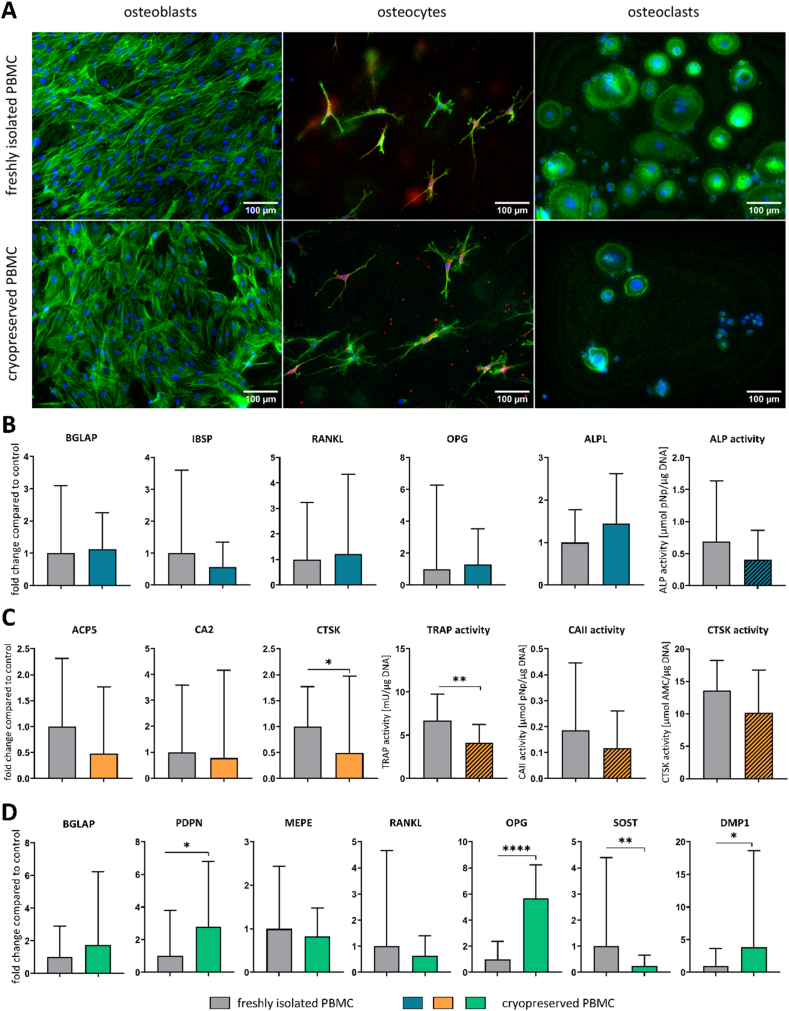
**OBs, OCys and OCs derived from freshly isolated PBMC or cryopreserved PBMC in triple culture. A**: Fluorescence microscopic images. Cytoskeleton appears green (iFluor488 phalloidin), nuclei appear blue (DAPI) and DMP1 appears red (Alexa546), scale bars represent 100 μm. **B**: Expression of OB-markers BGLAP, IBSP, RANKL, OPG and ALPL as well as ALP activity. **C**: Expression of OC-markers ACP5, CA2 and CTSK as well as TRAP, CAII and CTSK activities. **D**: Expression of OCy-markers BGLAP, PDPN, MEPE, RANKL, OPG, SOST and DMP1. Diagrams show fold changes normalized to triple cultures with freshly isolated PBMC ± upper and lower limit (each n = 6, in total n = 18), respectively mean values and standard deviation of enzyme activities (each n = 3, in total n = 9). High variances occur while combining three individual experiments due to variations between different donors. Therefore, gene expression data and enzyme activities of single experiments are shown in Figs. S3–5. *p < 0.05; **p < 0.01; ****p < 0.0001. (For interpretation of the references to colour in this figure legend, the reader is referred to the Web version of this article.)

OBs and OCys were differentiated from the same donor, enabling recovering of cells for repetition experiments from cryopreserved lots. In contrast, PBMC for OC-differentiation were freshly isolated prior to the experiment in our previous studies. In the present study (see 3.2., Fig. 4) we successfully differentiated OCs from cryopreserved PBMC in monoculture with the addition of RANKL and MCSF. Based on these findings we further investigated the use of fresh versus cryopreserved PBMC in triple culture experiments. Considering the results of the media optimisation for simultaneous differentiation of OCys and OCs, a triple culture model was established (Fig. 1). Morphology, gene expression and enzyme activities of OBs and OCys in triple culture with freshly isolated PBMC in comparison to cryopreserved PBMC were analysed (Fig. 5). OBs and OCys showed their typical morphology in triple culture with both cryopreserved and fresh PBMC. However, when cryopreserved PBMC were used to differentiate OCs in triple culture, the number of OCs was considerably reduced compared to freshly isolated PBMC (Fig. 5A). Gene expression analysis did not reveal any difference in expression of OB-markers and ALP activity (Fig. 5B), whereas OC-markers and corresponding enzyme activities tended to be reduced in triple culture with cryopreserved PBMC. Nevertheless, activities of three OC-specific enzymes TRAP, cathepsin K (CTSK) and carbonic anhydrase II (CA II) proved the functionality of generated OCs (Fig. 5C). CTSK expression and TRAP activity were significantly lower when cryopreserved and thawed PBMC were applied for the triple cultures. The expression of SOST in OCys was downregulated in triple culture with frozen PBMC, but PDPN and DMP1 as well as OPG were upregulated (Fig. 5D). In summary, cryopreservation of PBMC affected OCs and OCys, while OBs were rather indifferent to PBMC pretreatment.

### 3.4 Effect of sHA3 on advanced bone triple cultures in comparison to monocultures

**Fig. 6:**
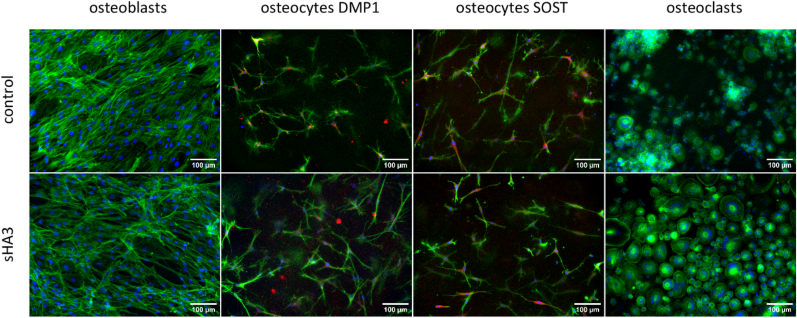
**Fluorescence microscopic images of OBs, OCs and collagen gel-embedded OCys in triple cultures in the presence and absence of sHA3.** Cytoskeleton appears green (iFluor488 phalloidin), nuclei appear blue (DAPI), DMP1 as well as SOST appear red, scale bars represent 100 μm. (For interpretation of the references to colour in this figure legend, the reader is referred to the Web version of this article.)

**Fig. 7:**
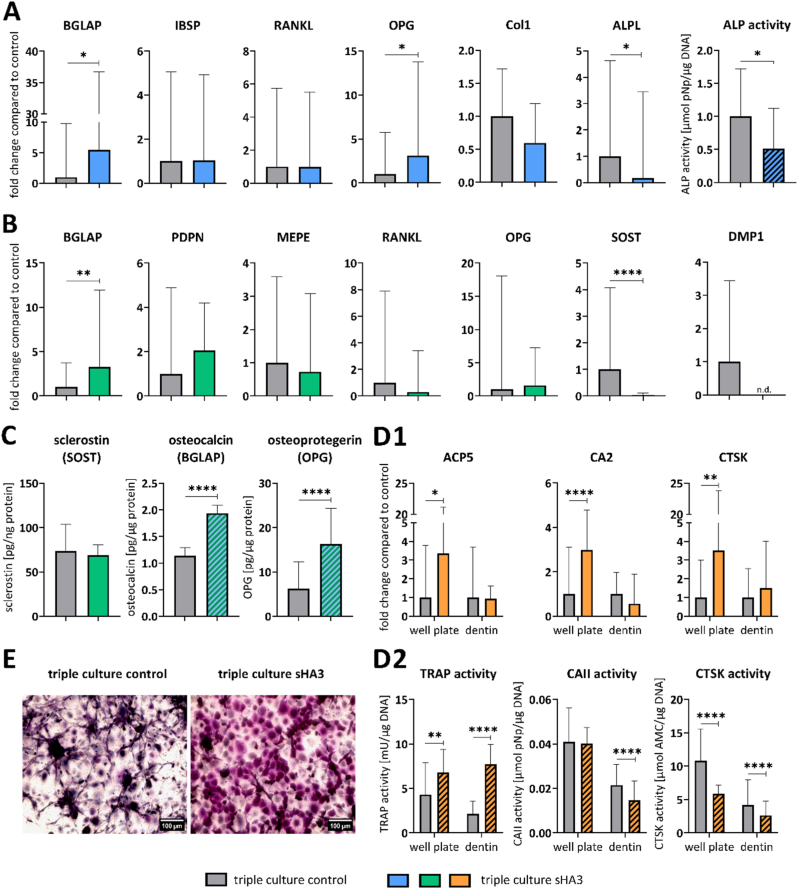
**OBs, OCys and OCs derived from PBMC in triple culture with and without sHA3**. A: Expression of OB-markers BGLAP, IBSP, RANKL, OPG, Col1 and ALPL as well as ALP activity. B: Expression of OCy-markers BGLAP, PDPN, MEPE, RANKL, OPG, SOST and DMP1. C: Quantification of secreted SOST, BGLAP and OPG in triple culture supernatants. D1: Expression of OC-markers ACP5, CA2 and CTSK on tissue culture polystyrene (TCPS) and on dentin slides. D2: TRAP, CAII and CTSK activities of OCs in well plates and on dentin slides. E: TRAP staining of OCs on TCPS, scale bars represent 100 μm. Diagrams show fold changes normalized to triple cultures without sHA3 ± upper and lower limit (each n = 6, in total n = 24), respectively mean values and standard deviation of enzyme activities (each n = 3, in total n = 12). High variances occur while combining four individual experiments due to variations between different donors. Therefore, gene expression data and enzyme activities of single experiments are shown in Figs. S6–9. *p < 0.05; **p < 0.01; ****p < 0.0001.

**Fig. 8:**
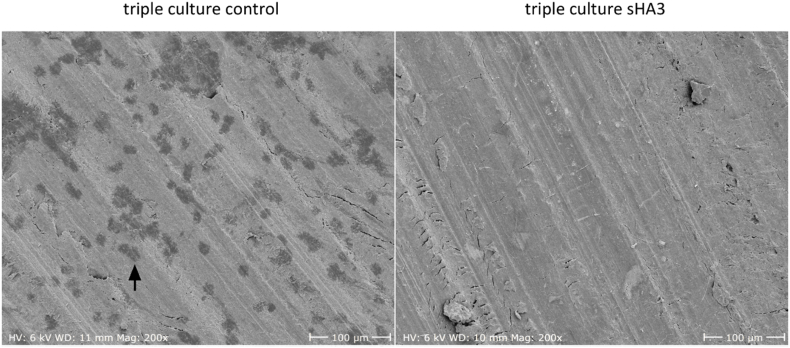
**Representative SEM images of dentin samples after osteoclastic resorption in triple culture after 14 days with and without sHA3.** Scale bars represent 100 μm. Resorption pit indicated by black arrow.

**Fig. 9:**
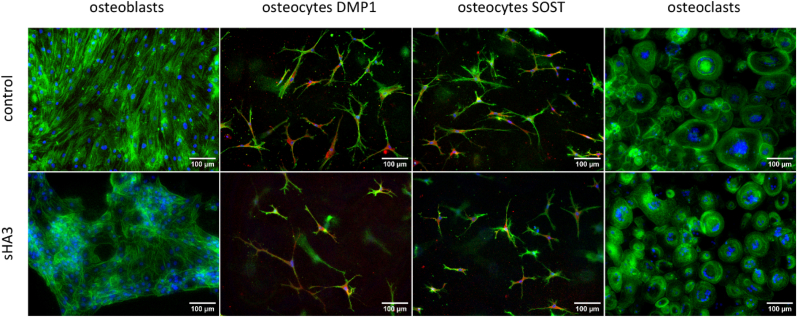
**Fluorescence microscopic images of monocultured OBs, OCs and collagen gel-embedded OCys in the presence and absence of sHA3.** Cytoskeleton appears green (iFluor488 phalloidin), nuclei appear blue (DAPI), DMP1 as well as SOST appear red, scale bars represent 100 μm. (For interpretation of the references to colour in this figure legend, the reader is referred to the Web version of this article.)

Cell morphology, gene expression, enzyme activities, protein secretion (BGLAP, SOST, RANKL, OPG) as well as resorption were analysed in triple cultures of OBs, OCys and OCs derived from freshly isolated PBMC in response to a treatment with 200 μg/mL synthetically derived sHA3 (Fig. 6, Fig. 7) compared to bone cell monocultures (Fig. 8, Fig. 9). All three bone cell species developed their typical morphology in triple culture, with and without addition of sHA3 (Fig. 6). OCy-specific DMP1 and SOST were detected in all groups, mainly located in the centre of the cells around the nucleus. However, number and size of multinucleated OCs were considerably higher in triple cultures with sHA3 treatment.

Gene expression and activity of OB-specific ALP was downregulated in the presence of sHA3, whereas OPG expression was upregulated (Fig. 7A). In response to sHA3, OPG expression was more dominant than RANKL expression (Table S1 RANKL/OPG ratio). BGLAP expression of OBs and OCys was significantly increased in the presence of sHA3 in triple culture. In contrast, OCy-specific SOST and DMP1 expression was strongly downregulated or undetectable in response to sHA3 (Fig. 7B). In line with the enhanced gene expression of BGLAP and OPG, respective protein levels in triple culture supernatants were significantly increased by sHA3 (Fig. 7C). The amount of secreted RANKL in triple culture supernatants was below the detection limit of the ELISA (<78.1 pg/mL). OC-markers were significantly upregulated in the presence of sHA3, when PBMC were cultured on tissue culture polystyrene (TCPS) in triple culture (Fig. 7D1). Likewise TRAP activity (Fig. 7D2) and TRAP staining (Fig. 7E) were strongly intensified with sHA3. Cathepsin K activity was reduced in response to sHA3.

PBMC seeded on dentin in triple culture were able to form resorption pits after 14 days as seen in Fig. 8 (resorption pit indicated by black arrow). Importantly, the treatment with sHA3 completely inhibited resorptive activity of the differentiated OCs.

In comparison to triple cultures, monocultures of OBs, OCys and OCs were exposed to sHA3. Monocultures were treated with the same medium composition as triple cultures, except for OC monocultures which received additional RANKL and MCSF. Morphology of OBs, OCys and OCs in monocultures was not impaired by sHA3 (Fig. 9, Fig. S13: enlarged image of osteoclasts from Fig. 9 to clearly demonstrate the multinucleated nature of the osteoclasts).

**Fig. 10:**
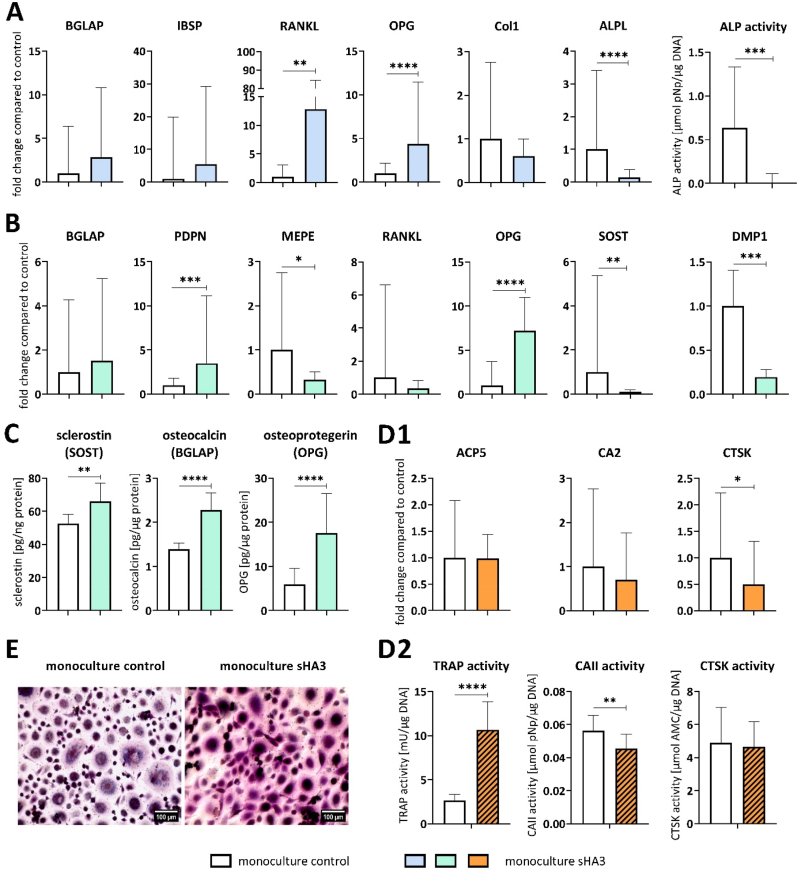
**Monocultured OBs, OCys and OCs derived from PBMC in the presence and absence of sHA3. A**: Expression of OB-markers BGLAP, IBSP, RANKL, OPG, Col1 and ALPL as well as ALP activity. **B**: Expression of OCy-markers BGLAP, PDPN, MEPE, RANKL, OPG, SOST and DMP1. **C**: Quantification of secreted SOST, BGLAP and OPG in OCy-supernatants. **D1**: Expression of OC-markers ACP5, CA2 and CTSK on TCPS. **D2**: TRAP, CAII and CTSK activities of OCs on tissue culture polystyrene. **E**: TRAP staining of OCs, scale bars represent 100 μm. Diagrams show fold changes normalized to monocultures without sHA3 ± upper and lower limit (each n = 6, in total n = 18), respectively mean values and standard deviation of enzyme activities (each n = 3, in total n = 9). High variances occur while combining three individual experiments due to variations between different donors. Therefore, gene expression data and enzyme activities of single experiments are shown in Figs. S10–12. *p < 0.05; **p < 0.01; ****p < 0.0001.

As for OB in triple culture, also for OB in monoculture, the ALP expression and activity were significantly reduced in the presence of sHA3 (Fig. 10A). BGLAP expression was not significantly changed in OB and OCy monocultures in the presence of sHA3. Osteocytic SOST, DMP1 and MEPE expression was downregulated, whereas PDPN and OPG were upregulated by sHA3 treatment of OCy monocultures (Fig. 10B). Secretion of BGLAP, OPG and SOST significantly increased in OCy supernatants treated with sHA3 (Fig. 10C). The amount of secreted RANKL in OCy supernatants was below the detection limit of the ELISA (<78.1 pg/mL). In contrast to triple cultures, OC-marker expression in monoculture on TCPS was not stimulated by sHA3 (Fig. 10D1). Instead, CTSK expression was downregulated in PBMC differentiated to OCs in monoculture. However, TRAP activity (Fig. 10D2) and TRAP staining (Fig. 10E, Fig. S14: enlarged image of osteoclasts from Fig. 10E to clearly demonstrate the multinucleated nature of the osteoclasts) were significantly increased in OC monocultures as in triple culture alike (Fig. 7D2, E), whereas CAII activity was reduced by sHA3.

**Fig. 11:**
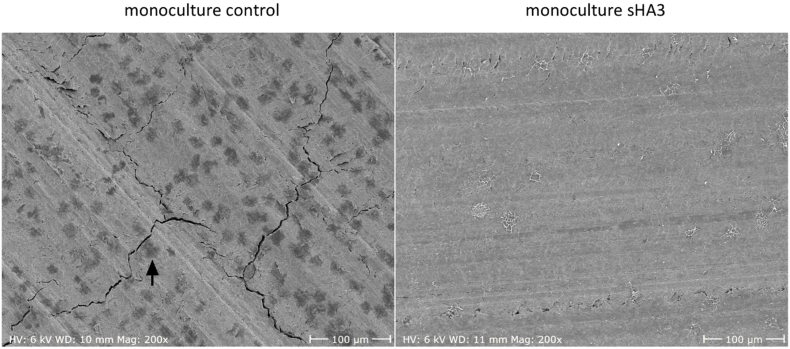
**Representative SEM images of dentin samples after osteoclastic resorption in PBMC monoculture after 14 days with and without sHA3.** Scale bars represent 100 μm. Resorption pit indicated by black arrow.

Monocultured OCs on dentin were able to form resorption pits after 14 days (Fig. 11, resorption pit indicated by black arrow). Like in triple culture, treatment with sHA3 completely inhibited the resorptive activity.

## 4 Discussion

### 4.1 Triple cultures with osteoblasts and simultaneously differentiating osteocytes and osteoclasts

The constant bone remodelling process is mediated by the action of bone forming OBs, bone resorbing OCs and regulating OCys. An *in vitro* model comprising these three cell types has recently been established in our group [3]. A limitation of this *in vitro* bone model is the differentiation of OCys and OCs in respective monocultures before assembling triple cultures. *In vitro* studies in bone cell monocultures cannot provide full insights into differentiation mechanisms since constant addition of supplements initialising differentiation, which are physiologically produced by the surrounding cells in bone tissue, is required. This may hinder the discovery of differentiation mechanisms based on cross talk between OBs, OCys and OCs. With the implementation of PBMC differentiating into OCs, direct effects on osteoclastogenesis as well as indirect effects via the secretion of cytokines like SOST and RANKL/OPG by OBs and OCys could be detected. To establish such a triple culture, media conditions are needed, allowing the simultaneous differentiation of OCys and OCs from their progenitors in the presence of OBs. Main obstacles are the different media requirements for OCy-differentiation (low serum) [25] and OC-formation from PBMC (10 % serum) [26].

OCy-differentiation was first optimised to achieve stable expression of markers such as DMP1, SOST and MEPE, which were not always present with OCy-medium containing 2 % FCS [3]. Gene expression analysis revealed an upregulation of BGLAP in the presence of vitamin D3 and a highly significant upregulation of SOST and RANKL by BMP-2. As described before, the active metabolite of vitamin D3: 1,25 (OH)2-vitamin D3 significantly increased BGLAP production in OBs [27] and BGLAP expression in induced pluripotent stem cell derived osteoprogenitors [28], making vitamin D3 a potent promoter of OB-OCy-transition. Induction of osteogenic differentiation with upregulated RANKL expression in response to BMP-2 was also reported by Ingwersen et al. [29]. In addition, SOST expression is known to be stimulated by BMPs in MLO-A5 osteocytes [30] as well as in Saos-2 cells [31]. Since Vitamin D3 increased BGLAP expression significantly, but not the expression of other OCy-markers, low serum containing media supplemented only with BMP-2 ensuring stable expression of OCy-marker genes, were further analysed for their ability to support osteoclastogenesis.

To evaluate OC-differentiation under low serum conditions, PBMC were differentiated into OCs in indirect co-culture with OBs under addition of human PL, ITS or reduced amounts of FCS compared to a 10 % serum control. Although some multinucleated OCs differentiated in all groups, TRAP activity was significantly reduced in low serum/alternative serum-based media. Wildt et al. also recently differentiated OCs from PBMC in the presence of 2.5 % PL [32] indicating possible formation of OCs under low serum conditions. Furthermore, OCs were differentiated from freshly isolated and cryopreserved PBMC. The use of cryopreserved PBMC instead of freshly isolated ones would allow repetition of experiments with the same donor combination and simplify the experimental setup, since PBMC would not have to be isolated freshly prior triple culture assembly. In PBMC monoculture with addition of RANKL, MCSF and 10 % serum, TRAP-positive OCs could be differentiated from fresh and cryopreserved PBMC. However, in ITS-containing media only few OCs differentiated, regardless of fresh isolation or cryopreservation of PBMC. The addition of BMP-2 did not impede formation of TRAP-positive OCs. Based on these results, a media composition that supports both OCy-differentiation and OC-differentiation in monoculture, containing 2 % serum (1 % hi FCS, 1 % HS) and BMP-2 was selected as triple culture medium. Triple cultures with OBs, collagen gel-embedded OBs and fresh/cryopreserved PBMC were conducted over 14 days. The *in vitro* bone model allows the separate analysis of the three primary human bone cell species without preventing interaction between the cells. Characteristics of OBs, OCys and OCs could be detected morphologically as well as on gene expression and protein level. However, when cryopreserved PBMC were used to differentiate OCs in triple culture, the number as well as marker expression and corresponding enzyme activities of OCs were considerably decreased. This suggests significant impact of the cryopreservation process on the functionality of PBMC to differentiate into OCs in triple culture. It has been reported that cryopreservation of PBMC can lead to reduced recovery and viability [33] as well as changes in cytokine secretion [34], which may explain the reduced potential of cryopreserved PBMC for OC-formation in triple culture. Expression of late OCy-marker SOST was downregulated in co-culture with cryopreserved and thawed PBMC, whereas early OCy markers PDPN and DMP1 were slightly upregulated. In conclusion, the improved triple culture is suitable to analyse simultaneous differentiation of OCys and OCs from their progenitors in the presence of OBs, however, PBMC should be isolated freshly.

### 4.2 Impact of sHA3 on bone cells in mono- and advanced triple cultures

GAGs have been extensively studied as potential agents for the functionalisation of bone graft materials. In previous studies, sHA3 enhanced osteogenic differentiation of human mesenchymal stem cells (hMSC) and pre-OBs [35]. On the other hand, sHA3 had an inhibitory effect on osteoclastogenesis and resorptive activity of osteoclasts [36]. However, mechanisms of action on the cellular level, in particular in a complex setting of co-cultured cells, are still not fully understood. Effects for cells in monoculture appear to be dependent on the degree of sulfation [16]. Therefore, both OB, OCy and OC monocultures as well as the established triple culture (see 4.1.) were exposed to synthetically derived sHA3 [37].

OB-specific ALP expression and activity was strongly downregulated in triple culture and OB monoculture in the presence of sHA3. Previously described pro-osteogenic effects of sHA derivatives [36] depend on the differentiation status of OBs: OB-precursor cells (hMSCs, rMSCs) and premature OBs respond to sHA treatment with significantly increased TNAP activity [38] compared to mature OBs [35]. In the present study, OBs pre-differentiated in osteogenic medium were used in triple culture representing mature OBs, which may explain the lack of pro-osteogenic effects. Schulze et al. recently found reduced TNAP-activity in response to sHA3 in OBs isolated from diabetes mellitus patients [19].

Interaction of sHA3 with BMP-2 prevents BMP-2 from binding to its receptor BMPR-IA [39]. As a result, the activity of BMP-2 as osteogenic supplement [29] is reduced, which explains the downregulation of ALP, SOST and DMP1 in triple culture and monocultures treated with sHA3. In conclusion, the binding of sHA3 to BMP-2 appears to neutralise pro-osteogenic effects of sHA3 and BMP-2.

In contrast, the expression and protein secretion of BGLAP was significantly upregulated in OBs and OCys in triple culture with sHA3, whereas BGLAP expression in OB- and OCy-monocultures only tended to be upregulated. Upregulation of BGLAP expression in response to sHA3 was also reported for monocultured murine MSC [36]. For OCys in triple culture, early markers like PDPN and BGLAP were upregulated, while late markers DMP1 and SOST were strongly downregulated in the presence of sHA3. BGLAP promotes the recruitment and differentiation of OC-precursors [40], suggesting involvement in OB-OC interaction and regulation of bone remodelling. This hypothesis is supported by an increased bone formation in BGLAP-deficient mice [41]. The stimulation of BGLAP on RNA and protein level after sHA3 treatment might contribute to the enhanced OC-formation and activity in triple culture.

Previously, it has been shown, that sHA3 suppressed OC-formation and inhibited OC-resorption in monoculture [18]. On the contrary, in triple culture, OC-marker expression, TRAP activity and TRAP staining were significantly increased in response to sHA3. Strong upregulation of OC-marker genes was only present in triple cultures, not in PBMC/OC monocultures, highlighting the importance of the triple culture model for evaluation of bioactive substances. Upregulation of TRAP activity in response to sHA3 was also recently reported by Schulze et al. [19] for monocultures of PBMC derived OCs, however the effect was only significant for PBMC, which were isolated from diabetic patients with a charcot neuropathy. Moreover, they found a decreased calcium phosphate resorption with sHA3, consistent with inhibited dentin resorption of OCs in triple and monoculture in response to sHA3. Cathepsin K activity in triple culture and CTSK expression in monoculture dropped with sHA3. These findings are in line with a decreased CTSK expression of RAW264.7 cells treated with sHA3 [18]. The protease cathepsin K degrades organic components of bone, such as collagen type I [42], which is responsible for bone resorption of OCs. Sulfated GAGs, can bind to triple helix type I collagen below pH 4.5 [43]. Interaction of sHA3 with collagen fibers may have protected them from cleavage by cathepsin K and inhibited dentin resorption by OCs. In summary the formation of osteoclast-like cells was stimulated by sHA3, while their maturation into functional resorbing osteoclasts was inhibited.

A reduced expression of SOST in OCys in the presence of high-sulfated GAG was previously reported [44]. Significantly downregulated SOST expression in triple culture and OCy-monoculture with sHA3 confirmed findings by Tsourdi et al.: Previous binding analyses showed a sulfate dependency of GAGs binding to SOST with increased binding strength through high sulfation [17,45]. OCy-produced SOST stimulates the expression of RANKL in OCys [46]. OC-differentiation is dependent on RANKL and MCSF [47]. OPG is a soluble RANKL decoy receptor that prevents OC-formation and osteoclastic bone resorption by inhibiting the interaction of RANKL with its receptor RANK, and is produced by OBs and OCys [48]. Binding of RANKL to its receptor RANK induces downstream signaling cascades that are essential for the transcription of OC-specific genes such as ACP5 and CTSK [49]. Since recombinant RANKL was added to PBMC monocultures for OC-differentiation, expression of OC-markers was less affected by sHA3 treatment. In triple culture, no external RANKL or MCSF was added to allow OC-differentiation only by signaling between OBs/OCys and PBMC. Even the treatment with sHA3 induced OPG on gene expression and protein level, but not SOST or RANKL expression, OC-formation and marker expression were significantly increased in triple culture, suggesting important mechanisms or targets of sHA3 in OC-differentiation. A treatment for 24 h with nearly doubled sHA3 concentration (389.44 μg/mL) compared to the triple culture experiment (200 μg/mL) also lowered the RANKL/OPG expression ratio in OB-like UMR-106 cells [17]. Salbach-Hirsch et al. showed an altered RANKL/OPG interaction in response to sulfated GAG derivatives [50]. Their hypothesis revealed direct binding of sHA3 to OPG, which prevents OPG from inhibiting RANKL/RANK interaction by electrostatic repulsion between GAGs and a negatively charged region in RANKL. As a result, RANKL/OPG ratio is increased, leading to enhanced OC-formation. Sulfation is essential for this OPG-blocking function of GAGs, as desulfated hyaluronan [50] or heparin loses its ability to bind and block OPG from binding to RANKL [51]. Since TRAP staining of OCs was strongly intensified in the presence of sHA3, even in PBMC monoculture with added RANKL and MCSF, where OPG was not present, alternative mechanisms of sHA3 in OC-differentiation besides RANKL/OPG signaling are suggested. Possibly, sHA3 itself can bind to RANK or other receptors on the surface of OCs and their precursors triggering OC-formation and activity. Another suggestion is the uptake of sHA3 through endocytosis and regulation of intracellular targets [36,52]. Further studies are needed to identify the factors mediating OC-formation, their activation and mechanisms of action to better understand the bone remodelling process and to identify potential targets for medical treatment of bone remodelling related diseases. Further experiments are required to fully understand mechanisms of action of sHA3 on the bone remodelling process. The established *in vitro* bone model is still not completely comparable to the clinical situation, as the impact of shear stress on OCys and the influence on angiogenesis and thus osteogenic-angiogenic coupling are not part of the model. Ongoing research aims to implement endothelial cells into the *in vitro* bone model.

## 5 Conclusions

A three-dimensional triple culture of primary human OBs and simultaneously differentiating OCys and OCs was successfully established. OCy-differentiation in collagen gels from primary human OBs was significantly improved by administration of BMP-2 ensuring a stable OCy-marker expression. OC-differentiation was achieved even under low serum conditions from both freshly isolated and cryopreserved PBMC in monoculture. Differentiation processes were induced by cellular signalling between the cell types without addition of external RANKL/MCSF, displaying a significant improvement to existing co-culture models, were bone cells differentiate prior their assembly to a co-culture. The established human *in vitro* bone model was applied to analyse the impact of sHA3 on the three bone cell species and their interaction in triple culture compared to OB/OCy/OC monocultures. Stimulating effects of sHA3 in triple culture were observed for the formation of osteoclast-like cells and their TRAP activity, but not for their actual resorptive activity and therefore functionality. OPG expression and protein secretion, but not RANKL or SOST, are strongly enhanced by sHA3, suggesting an important role of sHA3 itself in osteoclastogenesis with other targets than indirect modulation of RANKL/OPG ratio. BGLAP expression and protein secretion of OCys and OBs were upregulated in triple culture in response to sHA3, while ALP was downregulated. Binding of sHA3 to BMP-2 appears to decrease pro-osteogenic effects of both molecules, explaining the dropped ALP activity of OBs. This unique model allows to investigate the crosstalk between bone cell species as well as testing of versatile bone healing factors, biomaterials or drugs in terms of differentiation, gene expression, protein secretion and activity. Differences in cellular behaviour in triple culture compared to monocultures indicate the importance of more complex co-culture systems with primary cells for more physiologically relevant *in vitro* analyses, especially when looking into differentiation processes to reduce the need of ethically questionable and costly animal studies.

## Funding

This study was founded by the 10.13039/501100001659German Research foundation (project number 281673234). Furthermore, KW was awarded a scholarship funded by and in cooperation with the Saxon Ministry for Higher Education and Arts and the Studentenwerk Dresden. JB acknowledges the financial support from the Ministry of Education, Youth and Sports of the Czech Republic via project NANOBIO (Reg. No. CZ.02.1.01/0.0/0.0/17_048/0007421).

## CRediT authorship contribution statement

**Katharina Wirsig:** Writing – review & editing, Writing – original draft, Visualization, Validation, Methodology, Investigation, Formal analysis, Data curation, Conceptualization. **Jana Bacova:** Writing – review & editing, Methodology, Investigation, Data curation. **Richard F. Richter:** Writing – review & editing, Methodology. **Vera Hintze:** Writing – review & editing. **Anne Bernhardt:** Writing – review & editing, Supervision, Project administration, Methodology, Funding acquisition, Conceptualization.

## Declaration of competing interest

The authors declare that they have no known competing financial interests or personal relationships that could have appeared to influence the work reported in this paper.

## Data Availability

Data will be made available on request.
