# Supplementary material for: Cellular response of advanced triple cultures of human osteocytes, osteoblasts and osteoclasts to high sulfated hyaluronan (sHA3)
Source: Mater Today Bio. 2024 Feb 22;25:101006. doi: 10.1016/j.mtbio.2024.101006 (PMC10912908; doi:10.1016/j.mtbio.2024.101006)
Supplement: Multimedia component 1 [file mmc1.docx]

**Cellular response of advanced triple cultures of human osteocytes, osteoblasts and osteoclasts to high sulfated hyaluronan (sHA3)**

**Supplementary materials**

Figure S1 Marker gene expression of collagen gel- embedded OCys cultivated over 14 days in different low serum containing media (FCS, ITS, hi FCS, HS) in the presence and absence of BMP-2 in concentrations between 33,3 ng/mL and 150 ng/mL. Diagrams show fold changes compared to 2 % FCS-based medium, +/- upper and lower limit of three experiments for ITS-based media (grey, each experiment n=6) and two experiments for hi FCS/HS based media (green, each experiment n=6). * p < 0.05; ** p < 0.01, *** p < 0.001; **** p < 0.0001.


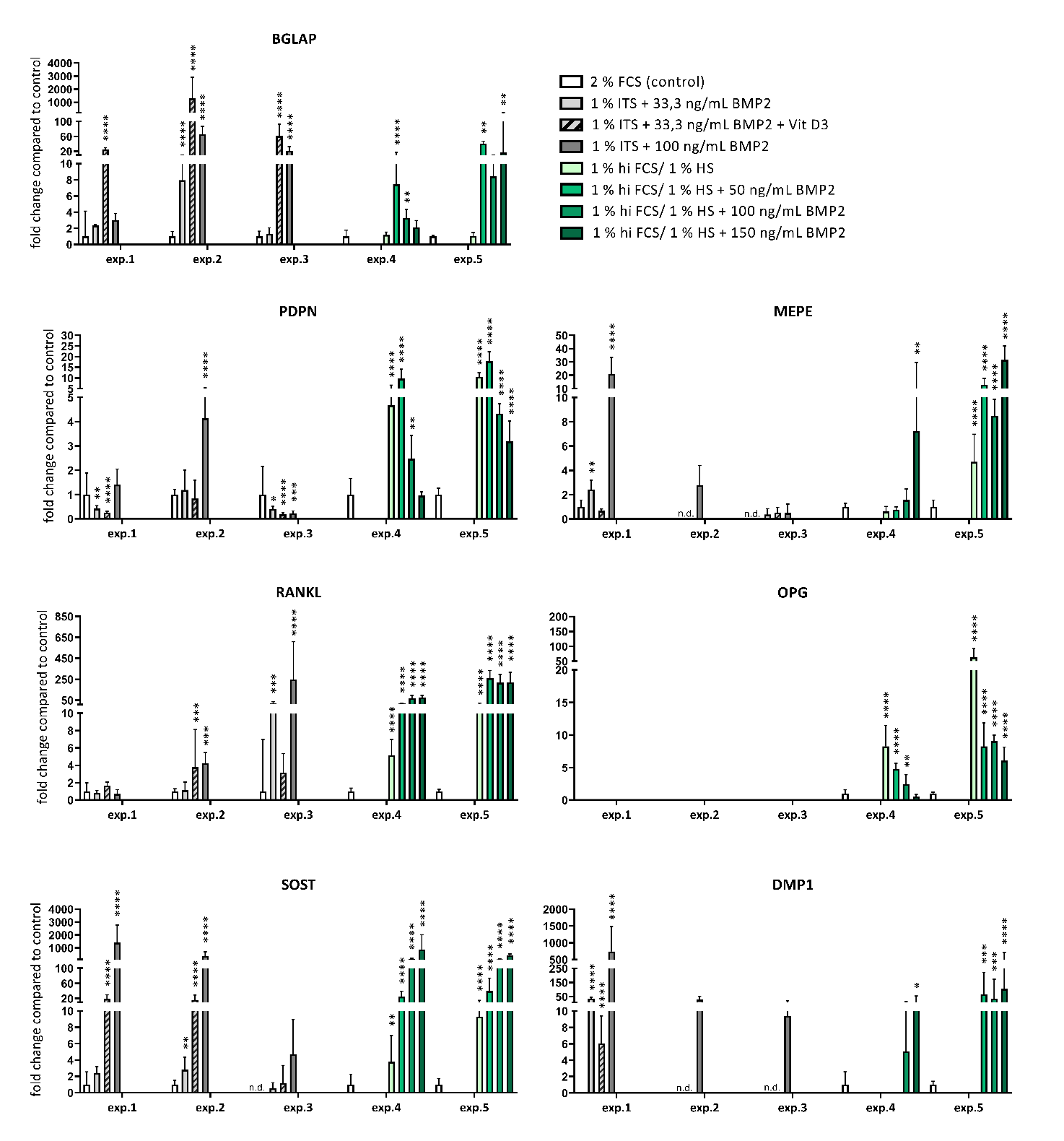


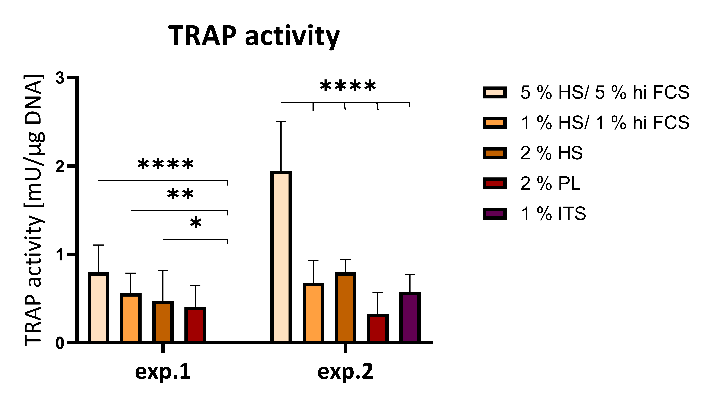


Figure S2 TRAP activity of OCs derived from freshly isolated PBMC in different low serum based media (hi FCS, HS, PL, ITS) in indirect co-culture with OBs. Diagram shows mean value +/- standard deviations of two individual experiments (each n=3). *** p < 0.001, **** p < 0.0001.


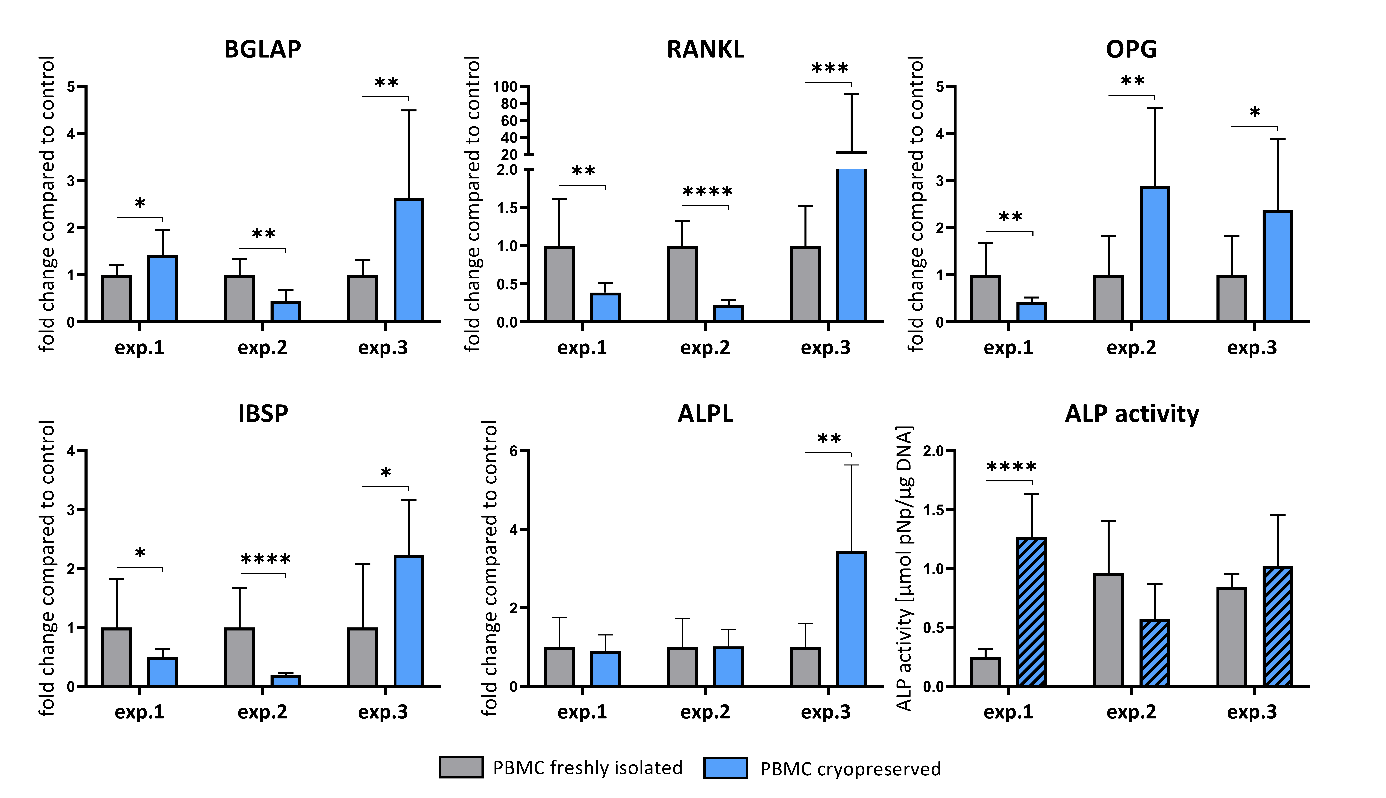


Figure S3 OBs in triple culture with OCys and OCs derived from freshly isolated and cryopreserved PBMC. Gene expression of OB-markers BGLAP, IBSP, RANKL, OPG and ALPL as well as ALP activity of three individual experiments with different donor combinations. Diagrams show fold changes compared to triple cultures with freshly isolated PBMC +/- upper and lower limit (each n=6), respectively mean values and standard deviation of enzyme activities (each n=3). * p < 0.05; ** p < 0.01; *** p < 0.001; **** p < 0.0001.


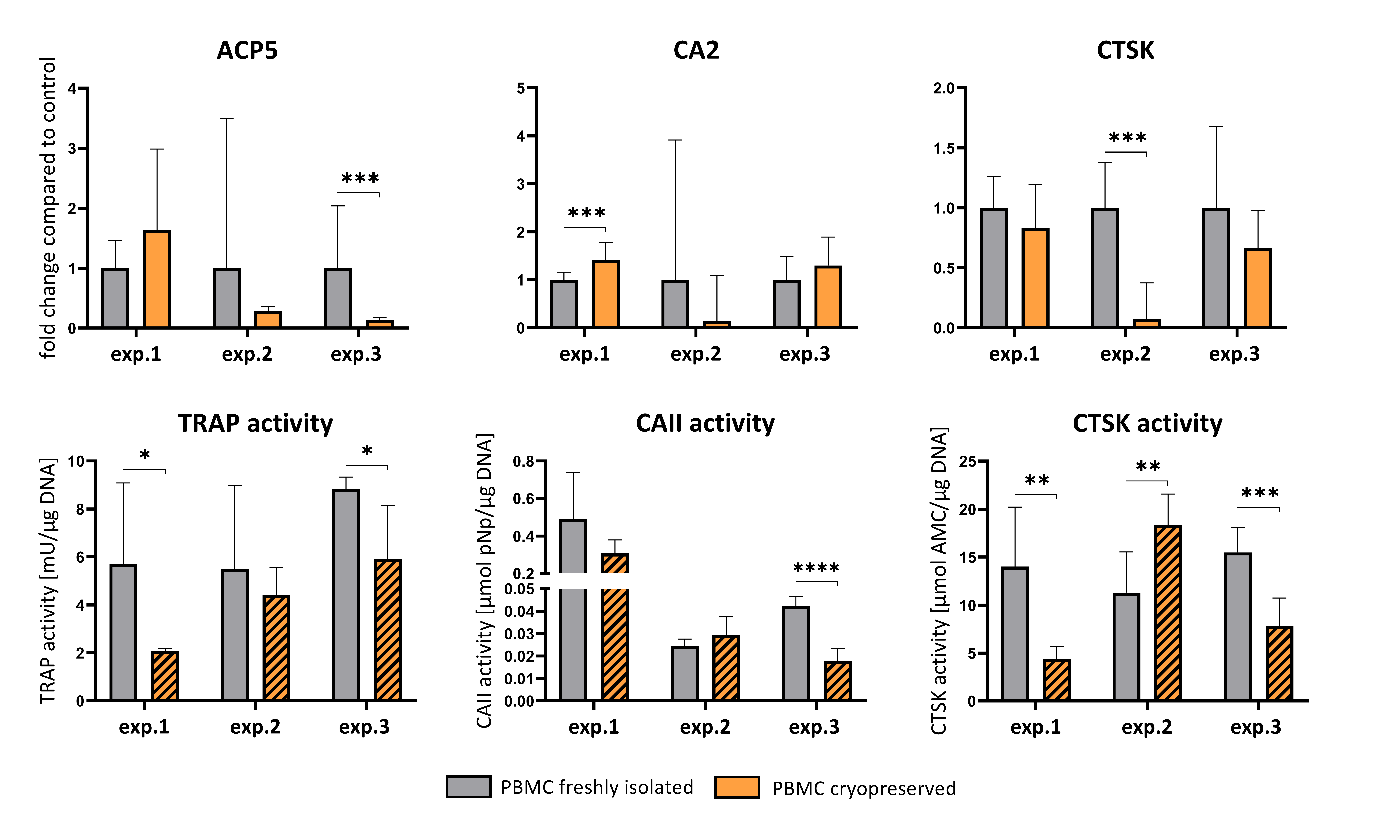


Figure S4 OCs derived from freshly isolated PBMC and cryopreserved PBMC in triple culture with OBs and OCys. Gene expression of OC-markers ACP5, CA2 and CTSK as well as TRAP, CAII and CTSK activities of three individual experiments with different donor combinations. Diagrams show fold changes compared to triple cultures with freshly isolated PBMC +/- upper and lower limit (each n=6), respectively mean values and standard deviation of enzyme activities (each n=3). * p < 0.05; ** p < 0.01; *** p < 0.001; **** p < 0.0001.

Figure S5 OCys in triple culture with OBs and OCs derived from freshly isolated and cryopreserved PBMC. Gene expression of OCy-markers BGLAP, PDPN, MEPE, RANKL, OPG, SOST and DMP1 of three individual experiments with different donor combinations. Diagrams show fold changes compared to triple cultures with freshly isolated PBMC +/- upper and lower limit (each n=6), * p < 0.05; ** p < 0.01; **** p < 0.0001.


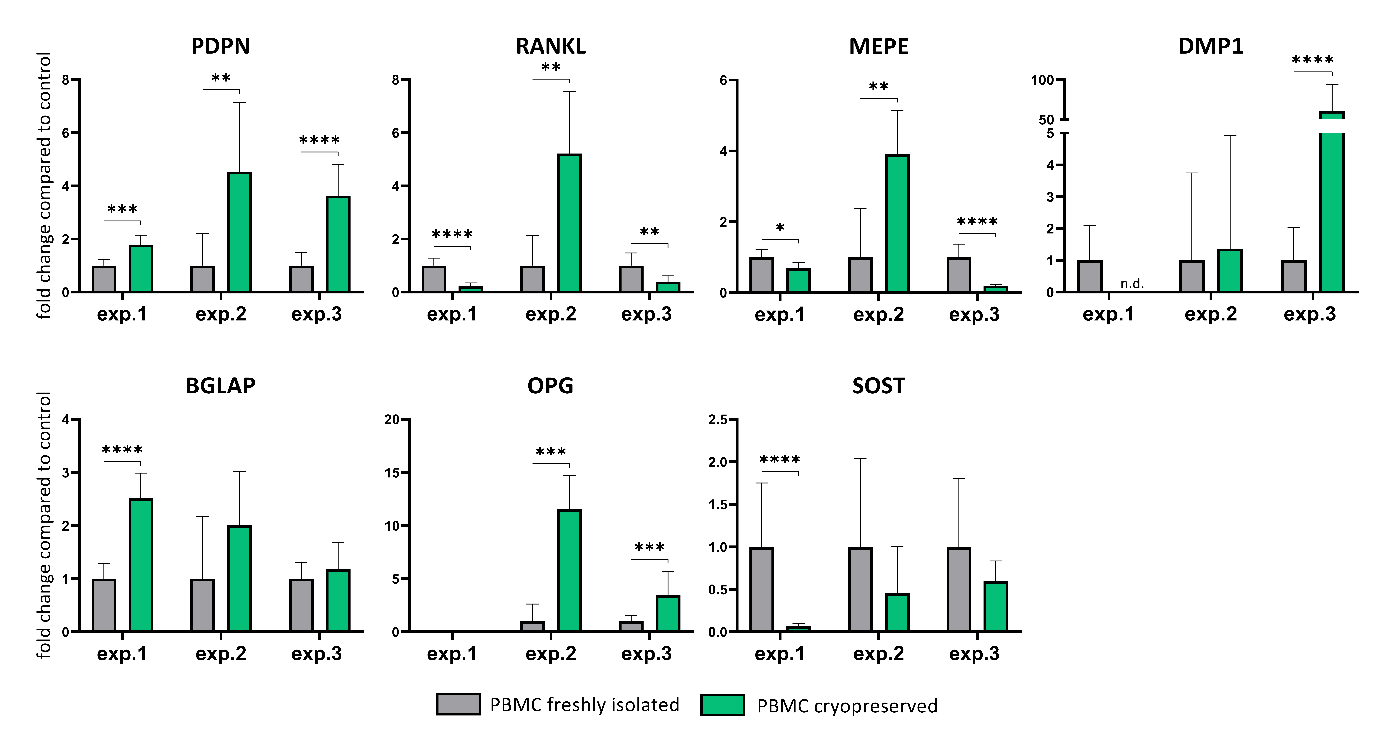


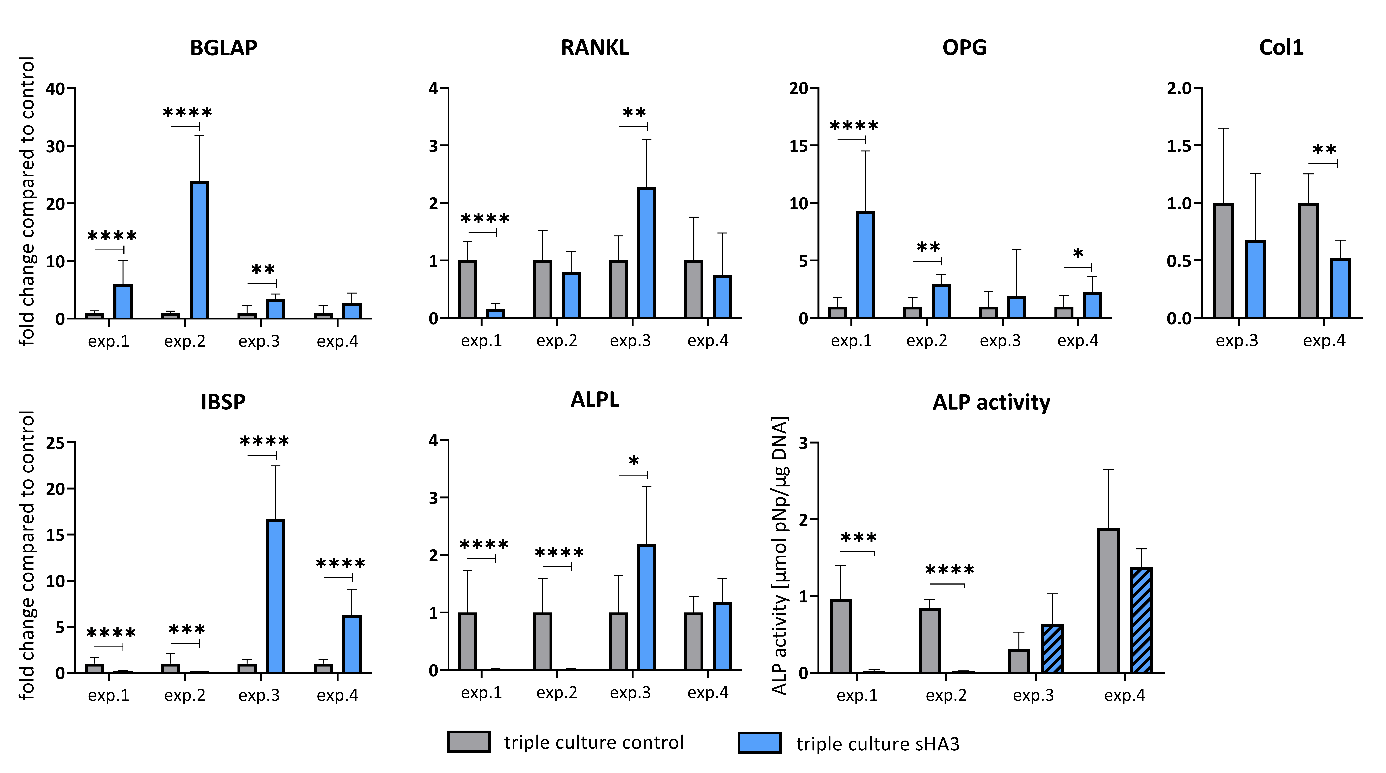


Figure S6 OBs in triple culture with OCys and OCs derived from PBMC with and without sHA3. Gene expression of OB-markers BGLAP, IBSP, RANKL, OPG and ALPL as well as ALP activity of four individual experiments with different donor combinations. Diagrams show fold changes compared to triple cultures without sHA3 +/- upper and lower limit (each n=6), respectively mean values and standard deviation of enzyme activities (each n=3). * p < 0.05; ** p < 0.01; *** p < 0.001; **** p < 0.0001.

Figure S7 OCs derived from PBMC on TCPS in triple culture with OBs and OCys with and without sHA3. Gene expression of OC-markers ACP5, CA2 and CTSK as well as respective enzyme activities of four individual experiments with different donor combinations. Diagrams show fold changes compared to triple cultures without sHA3 +/- upper and lower limit (each n=6), respectively mean values and standard deviation of enzyme activities (each n=3). * p < 0.05; ** p < 0.01; *** p < 0.001; **** p < 0.0001.


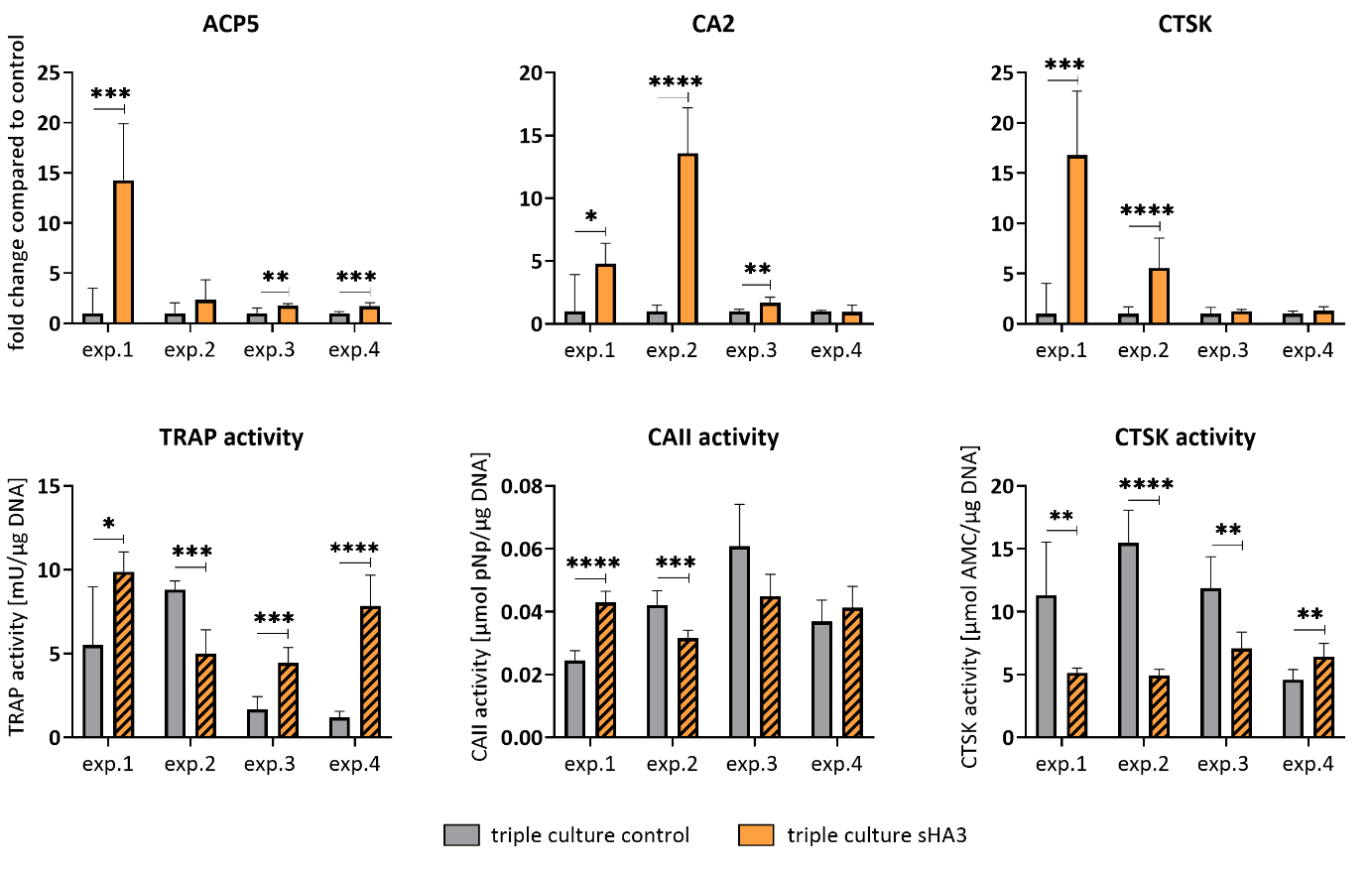


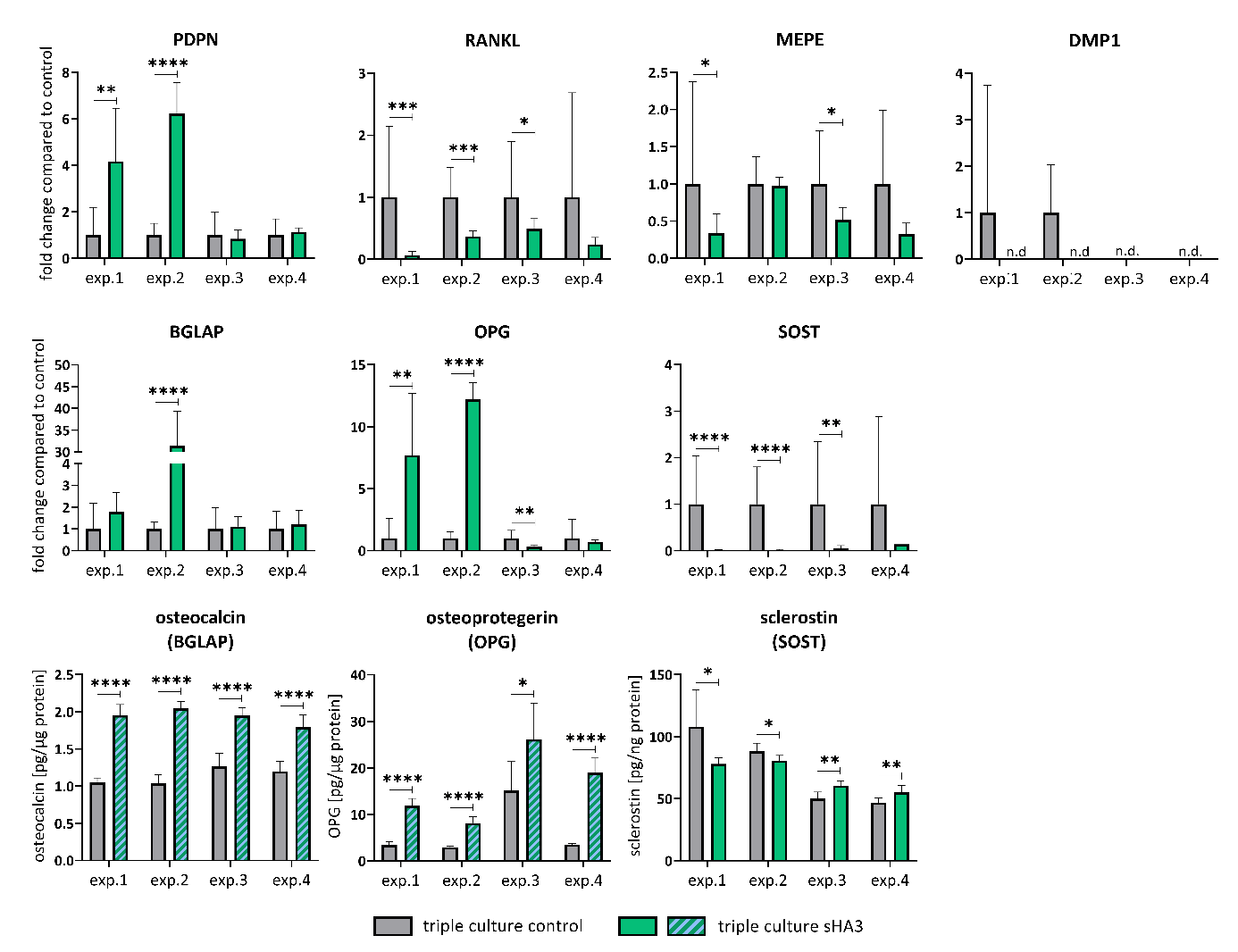

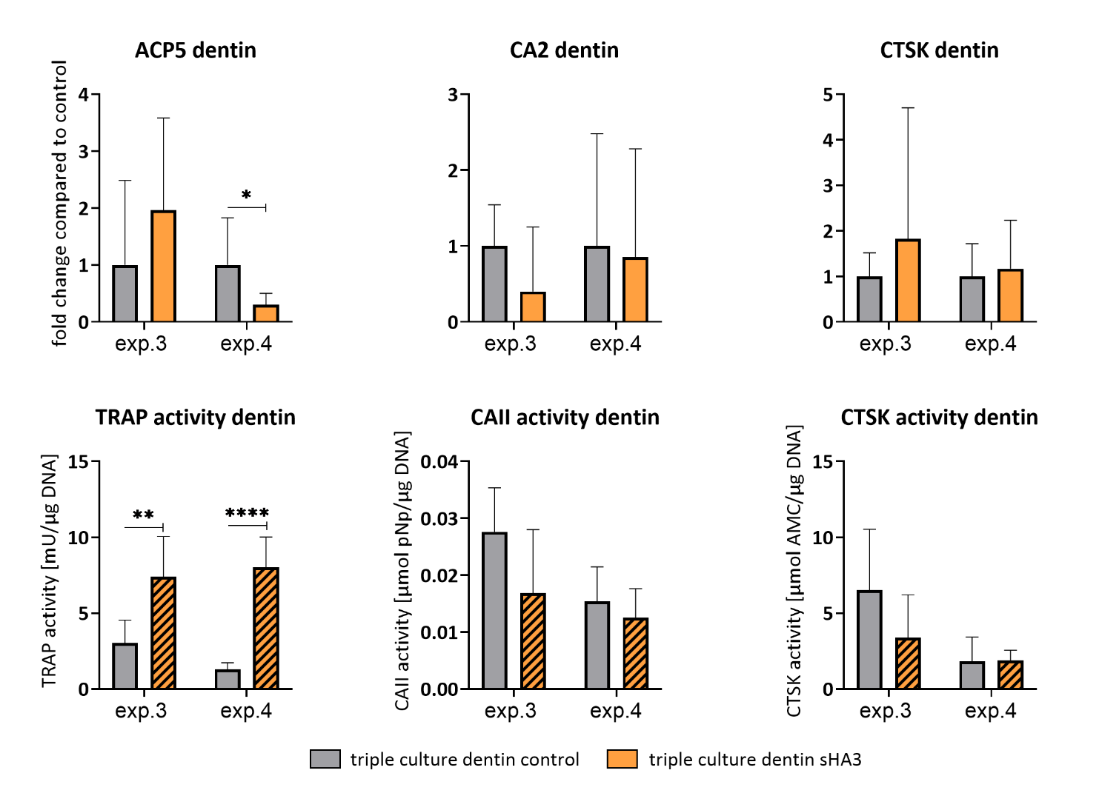


Figure S9 OCys in triple culture with OBs and OCs derived from PBMC with and without sHA3. Gene expression of OCy-markers PDPN, BGLAP, RANKL, OPG, MEPE, SOST and DMP1 as well as quantification of secreted SOST, BGLAP and OPG in triple culture supernatants of four individual experiments with different donor combinations. Diagrams show fold changes compared to triple cultures without sHA3 +/- upper and lower limit (each n=6), respectively mean values and standard deviation of secreted proteins (each n=3). * p < 0.05; ** p < 0.01; *** p < 0.001; **** p < 0.0001.

Figure S8 OCs derived from PBMC on dentin slides in triple culture with OBs and OCys with and without sHA3. Gene expression of OC-markers ACP5, CA2 and CTSK as well as respective enzyme activities of two individual experiments with different donor combinations. Diagrams show fold changes compared to triple cultures without sHA3 +/- upper and lower limit (each n=6), respectively mean values and standard deviation of enzyme activities (each n=3). * p < 0.05; ** p < 0.01; **** p < 0.0001.


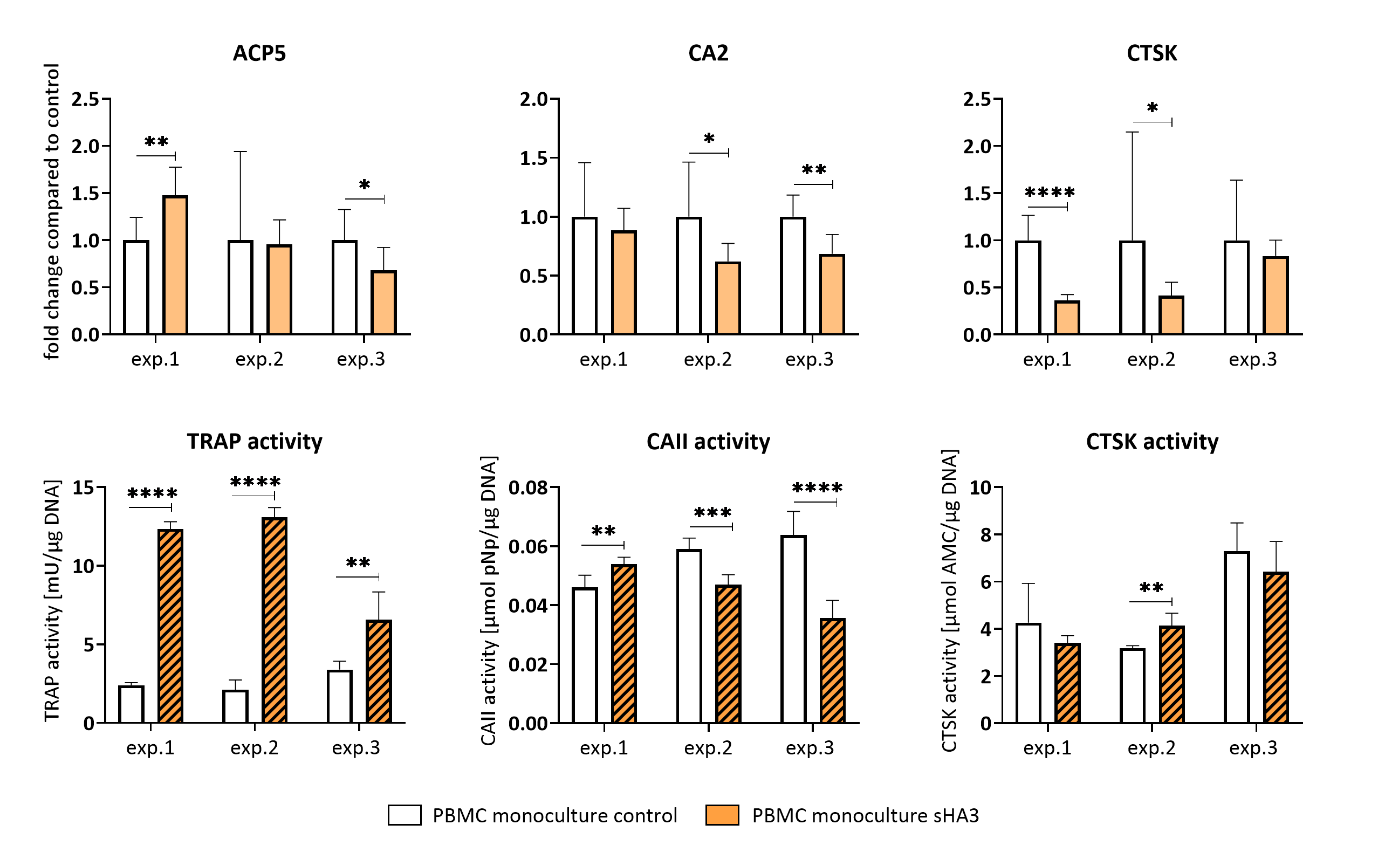

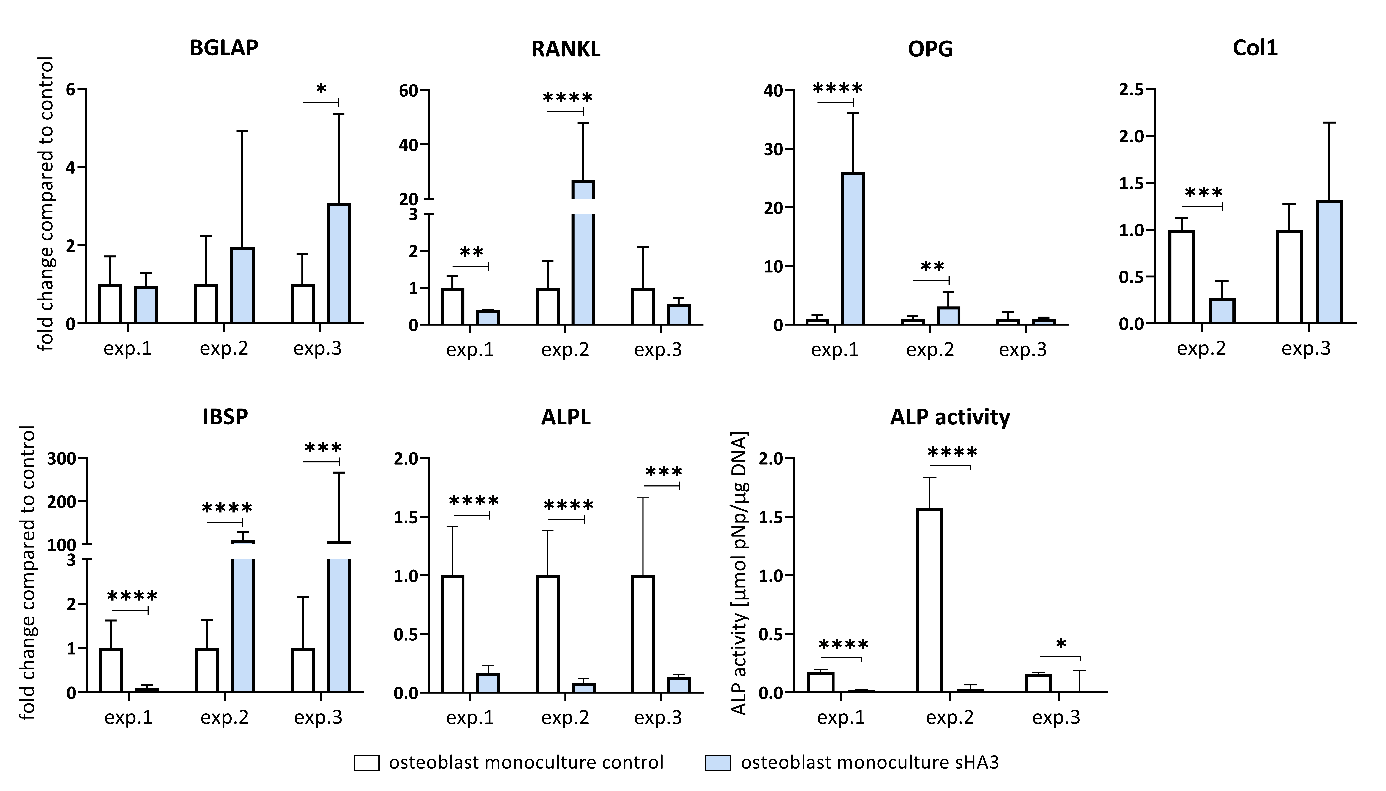


Figure S11 OC-monoculture derived from PBMC with and without sHA3. Gene expression of OC-markers ACP5, CA2 and CTSK as well as respective enzyme activities of three individual experiments with different PBMC donors. Diagrams show fold changes compared to OC-monoculture without sHA3 +/- upper and lower limit (each n=6), respectively mean values and standard deviation of enzyme activities (each n=3). * p < 0.05; ** p < 0.01; *** p < 0.001; **** p < 0.0001.

Figure S10 OB-monoculture with and without sHA3. Gene expression of OB-markers BGLAP, IBSP, RANKL, OPG, Col1 and ALPL as well as ALP activity of three individual experiments with different donors. Diagrams show fold changes compared to OB-monoculture without sHA3 +/- upper and lower limit (each n=6), respectively mean values and standard deviation of enzyme activities (each n=3). * p < 0.05; ** p < 0.01; *** p < 0.001; **** p < 0.0001.


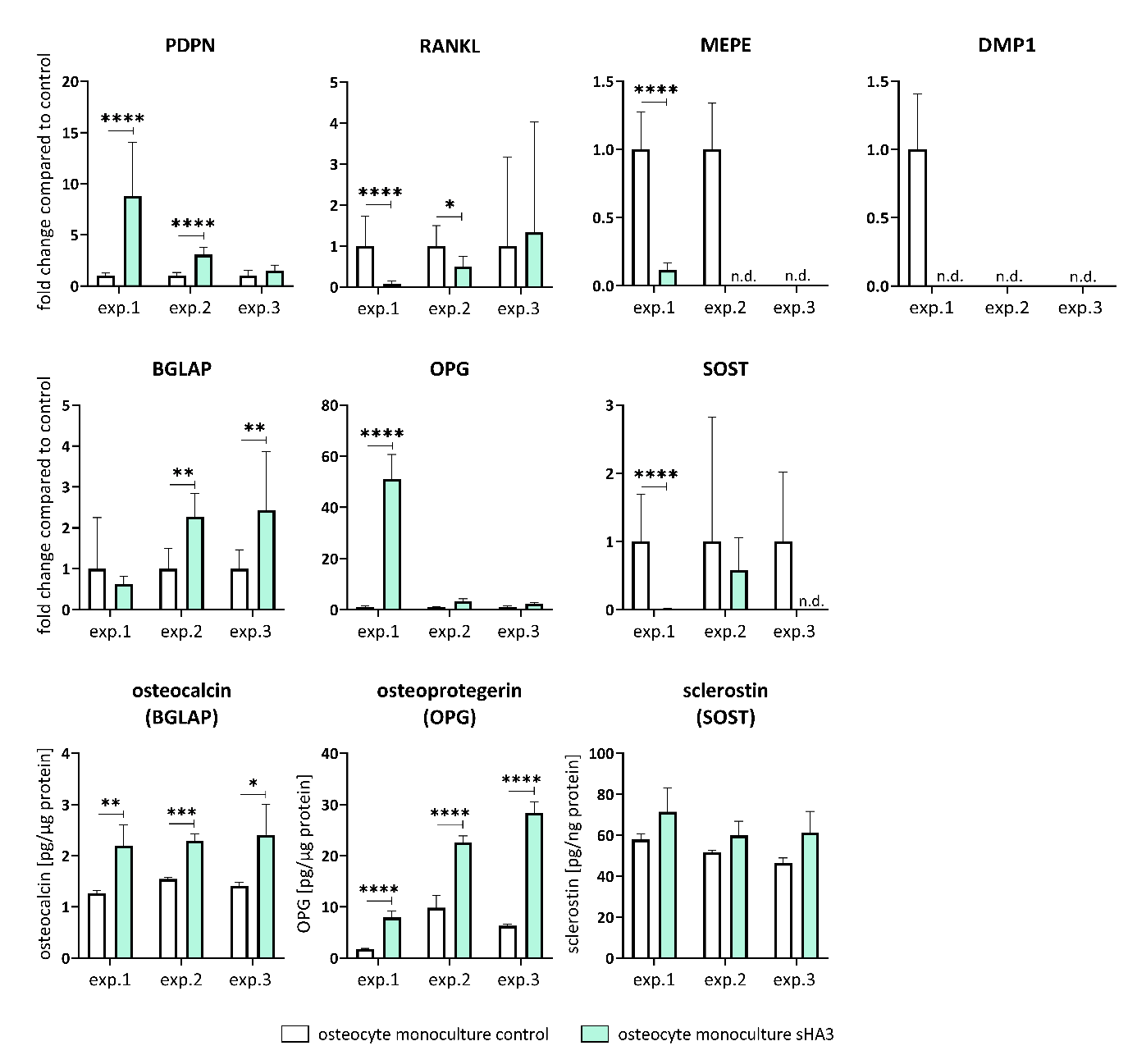


Figure S12 OCy-monoculture with and without sHA3. Gene expression of OCy-markers PDPN, BGLAP, RANKL, OPG, MEPE, SOST and DMP1 as well as quantification of secreted SOST, BGLAP and OPG in OCy-supernatants of three individual experiments with different donors. Diagrams show fold changes compared to osteocyte monoculture without sHA3 +/- upper and lower limit (each n=6), respectively mean values and standard deviation of secreted proteins (each n=3). * p < 0.05; ** p < 0.01; *** p < 0.001; **** p < 0.0001.


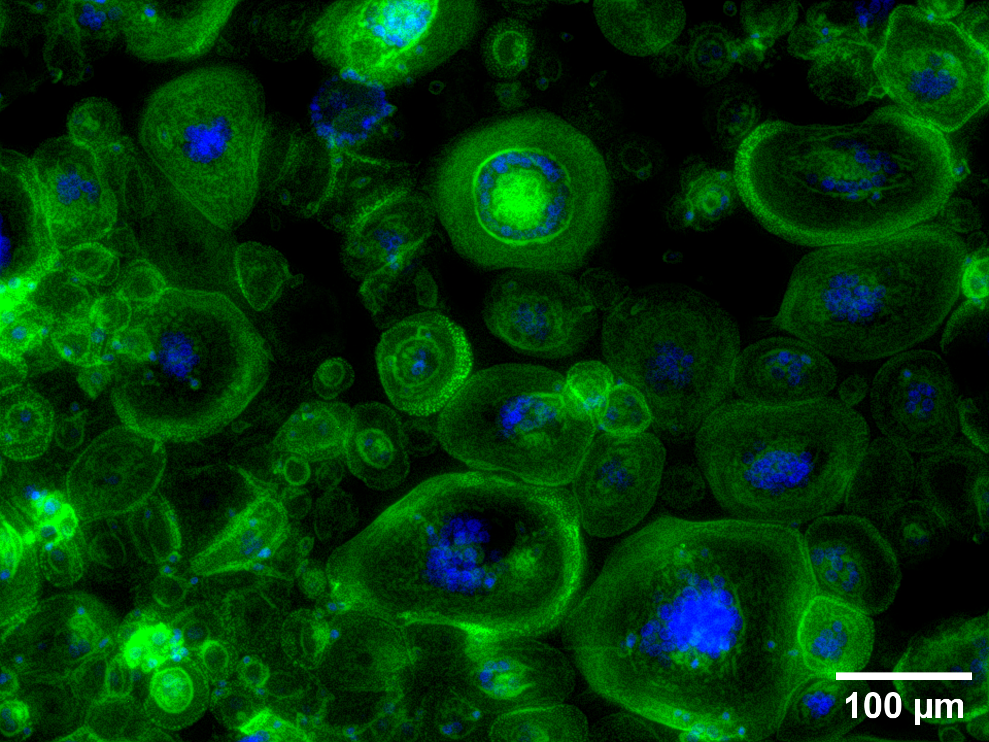


**Figure S13 Enlarged Figure 9 osteoclasts control**


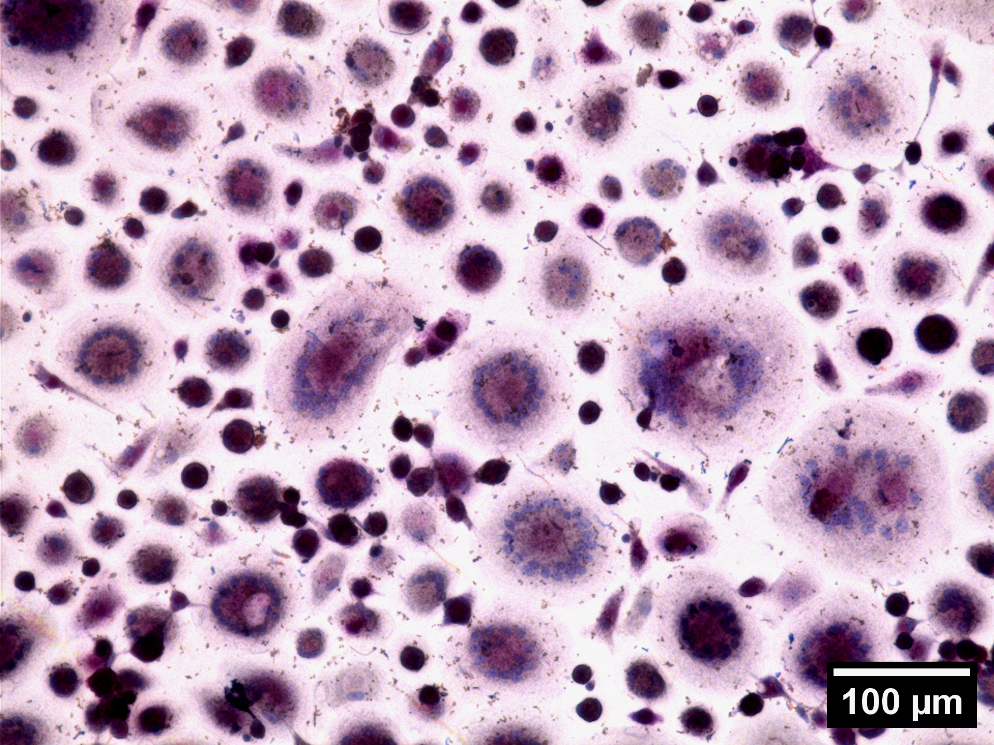


**Figure S14 Enlarged Figure 10E osteoclasts control**

**Table S1** Comparison of ΔCt values for RANKL and OPG expression as well as RANKL/OPG ratio of osteoblasts (OB) and osteocytes (OCy) in triple culture

| **ΔCt triple culture** | **RANKL** | **OPG** | **RANKL/OPG ratio** |
| --- | --- | --- | --- |
| OB control  OB sHA3  OCy control  OCy sHA3 | 8,71  8,74  9,60  11,31 | 3,46  1,81  4,79  4,11 | 2,52  4,82  2,00  2,75 |

**Table S2** Comparison of ΔCt values for RANKL and OPG expression as well as RANKL/OPG ratio of osteoblasts (OB) and osteocytes (OCy) in monoculture

| **ΔCt monoculture** | **RANKL** | **OPG** | **RANKL/OPG ratio** |
| --- | --- | --- | --- |
| OB control  OB sHA3  OCy control  OCy sHA3 | 12,07  8,39  13,18  14,67 | 4,41  2,29  5,38  2,53 | 2,74  3,66  2,45  5,80 |
